# An efficient lattice-based identity-based proxy Re-encryption scheme with direct revocation for cloud storage

**DOI:** 10.1371/journal.pone.0355279

**Published:** 2026-08-12

**Authors:** Juanjuan Li, Mingming Jiang, Yuyan Guo

**Affiliations:** School of Computer Science and Technology, Huaibei Normal University, Huaibei, China; China Medical University Taiwan, TAIWAN

## Abstract

As cloud storage becomes increasingly commonplace alongside the expansion of data sharing practices, how to achieve secure data sharing and user revocation while ensuring data confidentiality has become a key issue. Proxy re-encryption enables ciphertext originally designated for one user to be transformed by a semi-trusted proxy for another user, while identity-based encryption offers a simplified approach to key administration. However, most existing frameworks employ bilinear pairings, which suffer from susceptibility to quantum threats, and generally lack efficient revocation mechanisms. To tackle these challenges, we present an identity-based directly revocable proxy re-encryption scheme (IBPRE-DR) based on the Learning With Errors (LWE) assumption. This scheme enables data owners to directly revoke user permissions without requiring a key generation center (KGC) to frequently update user keys, supporting immediate and dynamic access control. The mechanism uses a complete subtree algorithm to achieve efficient revocation. Additionally, a dual-key mechanism distinguishes re-encryption keys from decryption keys, effectively resisting collusion attacks and further enhancing data security. Furthermore, the private key length for each user remains fixed, supporting multi-bit encryption. We present a formal definition of IBPRE-DR’s security, along with a standard-model security proof. To the best of our knowledge, this is the first lattice-based identity-based proxy re-encryption scheme that integrates direct user revocation under the standard LWE assumption. Both experimental results and theoretical analysis confirm the scheme’s effectiveness and feasibility.

## 1. Introduction

As cloud storage services become increasingly popular, data outsourcing has become a prevalent approach for both organizations and individual users [1]. However, placing confidential information on cloud platforms that are not fully trusted raises significant privacy and security concerns. To preserve the confidentiality of information, data owners commonly apply encryption techniques to their datasets prior to uploading them to external storage environments. This, in turn, introduces a new challenge: how to securely and flexibly share the encrypted data with other authorized users without performing decryption.

Proxy re-encryption (PRE) provides a powerful cryptographic primitive to address this challenge. The notion of PRE was first introduced by Blaze et al. [2]. In a PRE system, a semi-trusted proxy is allowed to transform a ciphertext encrypted under one user’s public key into a ciphertext that can be decrypted by another user, without learning any information about the underlying plaintext.

Identity-based proxy re-encryption (IB-PRE) further simplifies key management by eliminating the need for traditional public key certificates. Instead, a user’s identity string (e.g., email address or unique identifier) is used directly as their public key. According to the delegation direction, IB-PRE schemes can be divided into unidirectional and bidirectional constructions. Bidirectional schemes can generally be derived from unidirectional ones through standard transformations. Ateniese et al. [3] summarized the main features of PRE as follows:

Non-interactivity: A trusted authority does not need to generate the re-encryption key $rk_{id_{A}\to id_{B}}$, as it can be produced from the public keys of users *A* and *B*.

Proxy Transparency: Both the delegating party and the receiving user remain unaware of the proxy’s involvement in the re-encryption process.

Key Optimality: The length of user B’s private key does not increase with the number of delegations received, remaining fixed irrespective of how many re-encryption permissions are granted.

Collusion Resistance (also referred to as Master Private Key Security): Even under collusion attacks involving the proxy together with user B, user A’s private key remains secure from derivation.

Non-transitivity: In the unidirectional setting, it is infeasible for the proxy to further delegate decryption rights specifically, even with knowledge of both $rk_{id_{A}\to id_{B}}$ and $rk_{id_{B}\to id_{C}}$, it cannot derive $rk_{id_{A}\to id_{C}}$.

Singh et al. [4] presented the first IB-PRE scheme built upon lattices, which supports anonymity, bidirectionality, and multi-hop functionality. Kirshanova [5] presented the inaugural lattice-based PRE scheme supporting unidirectional delegation, featuring collusion resistance and non-interactivity. Subsequently, many proxy re-encryption designs have been put forward [6–12]. Wu et al. [13] presented a lattice-based multi-hop, unidirectional IB-PRE scheme that achieves functional separation between decryption and re-encryption key generation through a dual private key mechanism. Zhang et al. [14] proposed a scheme enabling quantum-safe cloud data sharing and fine-grained keyword retrieval. Wang et al. [15] introduced the first lattice-based revocable identity-based proxy re-encryption (RIB-VPRE) scheme. Wang et al. [16] introduced the first lattice-based revocable IBEET scheme for mobile cloud computing. Their scheme addresses key exposure risks caused by device loss, resists quantum attacks, and achieves both efficiency and security.

However, existing IB-PRE schemes generally lack efficient and direct user revocation mechanisms, which limits their applicability in dynamic and large-scale cloud environments. In practical systems, revocation refers to the ability to immediately invalidate a specific user’s decryption capability without modifying the underlying encryption algorithm or affecting other legitimate users, thereby enabling flexible and dynamic access control. Revocation mechanisms can be categorized into two types:

Direct Revocation: The revocation operation is performed by the sender while executing the encryption operation by explicitly specifying a revocation list (RL). This approach does not require the key generation center (KGC) to update keys on a regular basis. When id∈RL, the revoked user is immediately unable to decrypt the ciphertext, and the revocation takes effect instantly.

Indirect Revocation: The revocation operation requires the KGC to periodically publish key update information. Users who have not been revoked are required to periodically refresh their keys; otherwise, they will be unable to decrypt.

### 1.1. Related work

Revocable Encryption. User revocation is a fundamental requirement in secure cloud data sharing systems. Boldyreva et al. [17] proposed the first identity-based encryption (IBE) scheme with indirect revocation, reducing the complexity of key updates from linear to logarithmic. Subsequently, Shi et al. [18] introduced a directly revocable encryption scheme based on verifiable key-policy attributes, enabling more flexible revocation management. To support dynamic access control in cloud environments, Ge et al. [19] proposed an identity-based broadcast proxy re-encryption scheme with key revocation, allowing cloud servers to revoke users without relying on the data owner’s private key. Ge et al. [20] further incorporated direct revocation into attribute-based proxy re-encryption (ABE-PRE), while Li et al. [21] designed a fast revocable ABE scheme with data integrity protection. Although these schemes improve revocation efficiency, their security mainly relies on classical hardness assumptions such as integer factorization and discrete logarithms, which are vulnerable to quantum attacks.

Lattice-Based Revocable Encryption. To address the threat posed by quantum computing, lattice-based cryptography has emerged as one of the most promising post-quantum cryptographic paradigms. Chen et al. [22] proposed the first lattice-based revocable IBE scheme, extending revocation functionality into the post-quantum setting. Zhao et al. [23] further constructed a revocable attribute-based encryption scheme under the R-LWE assumption, while Huang et al. [24] developed a lattice-based integrated revocable IBE scheme supporting anonymity, decryption key exposure resistance, and online/offline encryption. More recently, Wang et al. [16] proposed a lattice-based revocable IBE with equality test (RIBEET) for mobile cloud computing, combining user revocation, equality testing, and adaptive security. Chen et al. [25] designed a revocable attribute-based encryption scheme with efficient cloud-assisted verification for smart healthcare applications, where verification tags are dynamically updated during revocation. Huang et al. [26] introduced an efficient lattice-based revocable ABE scheme resistant to decryption key exposure for cloud file sharing. These studies demonstrate the effectiveness of lattice techniques in achieving quantum-resistant revocation and access control.

Lattice-Based Proxy Re-Encryption and Cloud Data Sharing. In parallel, researchers have explored advanced lattice-based cryptographic mechanisms for secure cloud data sharing. Feng et al. [27] proposed a blockchain-assisted lattice-based attribute-based searchable encryption framework integrating threshold key management and on-chain revocation, enabling secure and auditable data sharing. To enhance accountability and revocation in proxy re-encryption systems, Lin et al. [28] developed a lattice-based traceable and directly revocable attribute-based proxy re-encryption scheme with fair verification for medical IoT environments. Ahmad et al. [29] proposed a quantum-safe multi-factor authentication protocol for cloud-assisted Medical IoT systems, focusing on authentication and key agreement rather than proxy re-encryption or data sharing mechanisms. Beyond encryption mechanisms, Ahmad and Jagatheswari [30] proposed PQ-ABS, a post-quantum aggregate blind signature scheme based on Module-LWE and Module-SIS assumptions for anonymous authentication in blockchain-enabled Internet of Medical Things (IoMT). Ahmad and Jagatheswari [31] further proposed a quantum-secure lightweight fuzzy-extractor-based user authentication scheme for the Internet of Medical Things. Constructed on the RLWE assumption, the scheme employs fuzzy extractors to tolerate biometric variations and provides efficient mutual authentication and key agreement with formal security verification. In addition, Ahmad and Jagatheswari [32] presented a quantum-safe mutual authentication scheme for Internet of Healthcare Things (IoHT) using blockchain, which integrates lattice-based cryptography with blockchain technology to establish secure and decentralized authentication. The scheme enhances resistance against quantum attacks while ensuring data integrity, traceability, and secure access in healthcare environments.

Although significant progress has been made in revocable encryption, lattice-based access control, and post-quantum cloud data sharing, several limitations remain. Most existing revocable lattice-based schemes rely on periodic key-update generation and distribution by the KGC, making revocation timeliness dependent on the availability of update information. Furthermore, many existing lattice-based constructions focus on IBE or ABE settings and do not support proxy re-encryption. Even among proxy re-encryption schemes, direct revocation and immediate access control remain largely unexplored in identity-based lattice settings. To address these limitations, we propose the first lattice-based identity-based proxy re-encryption scheme with direct revocation (IBPRE-DR). In our construction, the data owner maintains the revocation list and embeds revocation information directly into the encryption process, enabling immediate revocation without periodic key updates while preserving post-quantum security under the LWE assumption. A summary of recent revocable encryption and authentication schemes is provided in Table 1.

**Table 1:** Summary of recent revocable encryption and authentication schemes.

Scheme	Core method/technique	Limitations/challenges
**Ge et al. [19]**	Identity-based broadcast PRE with key revocation	(1) Indirect revocation via KGC updates (2) High KGC overhead *O*(*N*) (3) Not post-quantum secure
**Ge et al. [20]**	Attribute-based PRE with direct revocation	(1) Based on classical pairings (2) Not post-quantum secure
**Chen et al. [22]**	First lattice-based revocable IBE	(1) Indirect revocation only (2) Periodic KGC updates required (3) No PRE functionality
**Zhao et al. [23]**	R-LWE-based revocable ABE	(1) Indirect revocation (2) No PRE functionality
**Huang et al. [24]**	Lattice-based integrated revocable IBE with En-DKER	(1) Indirect revocation (2) No PRE functionality
**Wang et al. [16]**	Lattice-based revocable IBE with equality test (RIBEET)	(1) Indirect revocation (2) No PRE functionality (3) KGC update overhead O(rlog(N/r))
**Chen et al. [25]**	Revocable ABE with cloud-assisted verification	(1) Based on pairings and not quantum-safe (2) Indirect revocation
**Huang et al. [26]**	Efficient lattice-based revocable ABE against key exposure	(1) Indirect revocation (2) No PRE functionality
**Feng et al. [27]**	Blockchain-enabled lattice ABSE with instant revocation	(1) No PRE functionality (2) Centralized authority dependency
**Lin et al. [28]**	Lattice-based traceable and directly revocable ABPRE	(1) KGC overhead *O*(*N*)
**Ahmad and Jagatheswari [29]**	M-LWE-based multi-factor authentication for cloud-assisted MIoT	(1) No revocation support (2) No PRE functionality

### 1.2. Our contributions

We construct a dual-key mechanism based on the trapdoor sampling algorithm proposed by Micciancio [33]. In this design, each user is associated with two functionally independent keys, where one is used for generating re-encryption keys and the other is used for decryption. This separation ensures that re-encryption keys and decryption keys are derived from distinct trapdoor components, thereby preventing key leakage and achieving collusion resistance. Even if a proxy colludes with an authorized delegatee, only partial key information is available, which is insufficient to recover the delegator’s private key.

During the user management phase, a full binary tree with N leaf nodes is constructed, where each user is mapped to a unique leaf node. Key generation leverages the dual-key structure to derive the corresponding basis for both re-encryption and decryption operations. The re-encryption key is generated from one component of the trapdoor basis, while the decryption key is derived from the other component and is used for decrypting both original and re-encrypted ciphertexts.

To support efficient revocation, the KUNodes algorithm is applied to the binary tree to compute a minimal node set covering all non-revoked users. This allows users to verify their revocation status by checking whether their corresponding path intersects with the computed node set, avoiding the need for exhaustive user traversal. The construction also supports multi-bit encryption, while keeping the length of each user’s private key fixed regardless of the number of encrypted bits.

During the encryption phase, the data owner directly specifies RL. This enables immediate access control and avoids the need for a continuously online KGC, which is required in traditional indirect revocation schemes. Therefore, the proposed scheme is suitable for dynamic cloud environments.

## 2. Preliminaries

We use bold lowercase symbols (e.g., 𝐚) to denote column vectors and bold uppercase letters (e.g., 𝐀) to denote matrices. The discrete Gaussian distribution over a lattice *L* with standard deviation σ is denoted by $D_{\sigma }(L)$. For a given vector 𝐱, let ‖𝐱‖ be its $\ell_{2}$ norm. For a matrix $𝐒\in {\mathbb{Z}}^{k\times m}$, ‖𝐒‖ represents the maximum $\ell_{2}$ norm of its column vectors. The Gram–Schmidt orthogonalization of 𝐒 is denoted by $\tilde{𝐒}$, and $\|𝐒{\|}_{2}$ denotes its spectral norm. In this context, we have $\left\|\tilde{𝐒}\|\leq \|𝐒\|\leq \|𝐒{\|}_{2}\leq \sqrt{k}\|𝐒\right\|$ and $\left\|𝐒𝐱\|\leq \|𝐒{\|}_{\text{sup}}\|𝐱\right\|$. In addition, a vertical bar is used to indicate the horizontal concatenation of vectors or matrices (e.g., [𝐀∣𝐁]), and a comma to denote vertical concatenation (e.g., [𝐀,𝐁]). The main notation used throughout this paper is summarized in Table 2.

**Table 2:** Notation Table.

Notation	Description
λ	Security parameter
*q*	Prime modulus
*n*,*m*	Lattice dimensions
χ	Error distribution over ${\mathbb{Z}}_{q}$
${𝐀}_{0},{𝐀}_{1}$	Public random matrices
𝐆	Gadget matrix
${𝐓}_{{𝐀}_{0}}$	Trapdoor basis of ${𝐀}_{0}$
$id,id_{A},id_{B}$	User identities
${𝐅}_{id}$	Identity-related public matrix
${sk}_{id}$	Private key associated with identity *id*
${rk}_{A\to B,y}$	Re-encryption key associated with node *y*
RL	Revocation list
BT	Binary tree for revocation management
Path(id)	Path associated with identity *id* in BT
KUNodes(BT,RL)	Covering node set for non-revoked users
μ	Message vector
${𝐂}_{id_{A}}$	Original ciphertext under identity $id_{A}$
${𝐂}_{id_{B}}$	Re-encrypted ciphertext for identity $id_{B}$
TrapGen,SamplePre	Trapdoor generation and preimage sampling algorithms
SampleLeft,SampleRight,SampleRightExtend	Lattice sampling algorithms

### 2.1. q-ary lattice

**Definition 1.** For positive integers *m*,*n* and *q* a prime modulus. Given $𝐀\in {\mathbb{Z}}_{q}^{n\times m}$ and $𝐮\in {\mathbb{Z}}_{q}^{n}$, the corresponding *q*-ary lattices are given as follows:


$$\begin{array}{cc} & \Lambda_{q}(𝐀)=\{𝐞\in {\mathbb{Z}}^{m}:\exists 𝐬\in {\mathbb{Z}}_{q}^{n}\text{ s.t. }{𝐀}^{⊤}𝐬=𝐞\;(\mathrm{mod}\;q)\},\\ & \Lambda_{q}^{⟂}(𝐀)=\{𝐞\in {\mathbb{Z}}^{m}:𝐀𝐞=0\;(\mathrm{mod}\;q)\},\\ & \Lambda_{q}^{𝐮}(𝐀)=\{𝐞\in {\mathbb{Z}}^{m}:𝐀𝐞=𝐮\;(\mathrm{mod}\;q)\}. \end{array}$$

If $𝐲\in \Lambda_{𝐪}^{u}(𝐀)$, then $\Lambda_{𝐪}^{u}(𝐀)=\Lambda_{q}^{⟂}(𝐀)+𝐲$; that is, $\Lambda_{𝐪}^{u}(𝐀)$ can be viewed as a shifted version of $\Lambda_{q}^{⟂}(𝐀)$.

**Lemma 1. ([**2,34**])**. For *q* > 2 and $𝐀\in {\mathbb{Z}}_{q}^{n\times m}$. Let ${𝐓}_{𝐀}$ be a short basis of the lattice $\Lambda_{q}^{⟂}(𝐀)$. Suppose $\sigma \geq \|\tilde{{𝐓}_{𝐀}}{\|}_{\text{GS}}\cdot \omega (\sqrt{\log m})$, and let $𝐮\in {\mathbb{Z}}_{q}^{n}$ and $𝐃\in {\mathbb{Z}}_{q}^{n\times m}$. It follows that:

1.
$\mathrm{Pr}\left[𝐱\leftarrow D_{\Lambda_{q}^{𝐮}(𝐀),\sigma }:\|𝐱\|>\sigma \sqrt{m}\right]\leq \mathrm{negl}(n);$

2. There is a PPT algorithm $SamplePre(𝐀,{𝐓}_{𝐀},𝐃,\sigma )$ which generates a matrix $𝐗\in \Lambda_{q}^{𝐃}(𝐀)$ (i.e., 𝐀𝐗≡𝐃(modq)) such that the distribution of 𝐗 is statistically close to $D_{\Lambda_{q}^{𝐃}(𝐀),\sigma };$
3.
$\mathrm{Pr}\left[𝐑\leftarrow D_{\Lambda_{q}^{𝐃}(𝐀),\sigma }:\|𝐑{\|}_{2}>m\cdot \sigma \right]\leq \mathrm{negl}(n);$

4.
$\mathrm{Pr}\left[𝐒\leftarrow \{-1,1{\}}^{m\times m}:\|𝐒{\|}_{2}>20\sqrt{m}\right]\leq \mathrm{negl}(n).$

### 2.2. Computational assumptions

**Definition 2. (LWE Hardness Assumption [**35–38**]**) Let *q* denote a prime modulus, an integer *n* satisfying n≥1, and a distribution χ defined on ${\mathbb{Z}}_{q}$. An instance of the ${LWE}_{n,q,\chi }$ problem is specified via a challenge oracle 𝒪 that the adversary can query, which operates either as a noise-injected pseudorandom oracle ${𝒪}_{𝐬}$ parameterized by a secret vector $𝐬\in {\mathbb{Z}}_{q}^{n}$, or as a uniformly random oracle ${𝒪}_{\$}$. Concretely:

- ${𝒪}_{𝐬}$ generates sample pairs of the form $({𝐮}_{i},v_{i})=({𝐮}_{i},{𝐮}_{i}^{⊤}𝐬+e_{i})\in {\mathbb{Z}}_{q}^{n}\times {\mathbb{Z}}_{q}$, the secret vector $𝐬\in {\mathbb{Z}}_{q}^{n}$ is selected uniformly at random, each ${𝐮}_{i}\in {\mathbb{Z}}_{q}^{n}$ is independently sampled from the uniform distribution, and the noise term $e_{i}\in {\mathbb{Z}}_{q}$ is taken from χ.
- ${𝒪}_{\$}$: Uniformly random instances in ${\mathbb{Z}}_{q}^{n}\times {\mathbb{Z}}_{q}$ are generated by ${𝒪}_{\$}$.

The search-LWE problem asks the adversary to recover the secret vector *s* given a polynomial number of samples from $O_{s}$. The decision-LWE problem asks the adversary to distinguish the sample pairs generated by $O_{s}$ from those generated by *O*_$_.

**Lemma 2. (Leftover Hash Lemma over LWE [39]**). Consider a prime modulus *q* > 2 and the condition m>(n+1)logq+ω(logn). Here, *k* = *k*(*n*) is taken as a polynomial. Let the matrices $𝐔\in \{-1,1{\}}^{m\times k}$, $𝐀\in {\mathbb{Z}}_{q}^{n\times m}$, and $𝐁\in {\mathbb{Z}}_{q}^{n\times k}$ be chosen uniformly at random. Subsequently, given an arbitrary vector $𝐯\in {\mathbb{Z}}_{q}^{m}$, the subsequent distributions are computationally indistinguishable:


$$\begin{array}{c} (𝐀,𝐀𝐔,{𝐔}^{⊤}𝐯)\;\text{and}\;(𝐀,𝐁,{𝐔}^{⊤}𝐯). \end{array}$$

**Lemma 3. (Worst-Case Hardness of LWE [18]**). If the LWE_*n,q,χ*_ problem admits an efficient distinguisher under the parameter constraints $q/B\geq 2n^{\varepsilon }$ for some function *B* = *B*(*n*) and *m* = poly(*n*), then one can construct a quantum algorithm for the worst-case SIVPγ problem, as well as a classical algorithm for ${GapSIVP}_{\gamma }$, where the approximation factor is given by $\gamma =2^{\Omega (n^{\varepsilon })}$.

### 2.3. Trapdoor and sampling algorithms

**Definition 3.(G-matrix) [**33,40,41**]**. For integer parameters satisfying q≥2 and n≥1, we define l=⌈logq⌉. There exists a structured matrix $𝐆={𝐈}_{n}⊗{𝐠}^{⊤}\in {\mathbb{Z}}_{q}^{n\times ln}$, where $𝐠=(1,2,...,2^{l-1})\in {\mathbb{Z}}_{q}^{l}$. A public basis ${𝐓}_{G}\in {\mathbb{Z}}^{ln\times ln}$ exists for the lattice $\Lambda_{q}^{⟂}(𝐆)$, with the property that $\|\tilde{{𝐓}_{G}}\|\leq \sqrt{5}$. Additionally, a deterministic function ${𝐆}^{-1}:{\mathbb{Z}}_{q}^{n\times ln}\to \{0,1{\}}^{ln\times ln}$ is defined, satisfying $𝐆\cdot {𝐆}^{-1}(𝐀)=𝐀$ and $\|{𝐆}^{-1}(𝐀){\|}_{2}\leq \ln$ for every $𝐀\in {\mathbb{Z}}_{q}^{n\times ln}$. Moreover, if m≥ln, the matrix 𝐆 can be expanded into $\overset{―}{𝐆}\in {\mathbb{Z}}_{q}^{n\times m}$ by adding zero columns on the right.

**Definition 4.(G-trapdoor) [**33,40,41**]**. Let integers *q* and *n* satisfy q≥2 and n≥1, and define $k=\lceil \log_{2}q\rceil$, *w* = *nk*, and $m=O(n\log_{2}q)$. Consider matrices $𝐀\in {\mathbb{Z}}_{q}^{n\times m}$ and $𝐆\in {\mathbb{Z}}_{q}^{n\times w}$ satisfying m≥w≥n. The 𝐆-trapdoor corresponding to 𝐀 takes the form of a matrix $𝐑\in {\mathbb{Z}}^{(m-w)\times w}$, an invertible matrix $𝐇\in {\mathbb{Z}}_{q}^{n\times n}$ for which the following condition holds:


$$\begin{array}{c} 𝐀[\begin{array}{c} 𝐑\\ {𝐈}_{w} \end{array}]=𝐇𝐆, \end{array}$$

where 𝐇 is the label (or tag) associated with the trapdoor.

**Lemma 4. (**[39,41]). For integers n≥1, q≥2, and m≥⌈2nlogq⌉, there exists a PPT algorithm $TrapGen(1^{n},1^{m},q)$ that produces a full-rank matrix $𝐀\in {\mathbb{Z}}_{q}^{n\times m}$ together with a short basis ${𝐓}_{𝐀}\in {\mathbb{Z}}^{m\times m}$ for the lattice $\Lambda_{q}^{⟂}(𝐀)$. Furthermore, the resulting matrix 𝐀 lies statistically near uniform, and the Gram–Schmidt norm of ${𝐓}_{𝐀}$ meets the bound $\left\|\tilde{{𝐓}_{𝐀}}\|\leq O(\sqrt{n\log q}\right)$.

The polynomial-time algorithm $SampleBasisLeft(𝐀,𝐁,{𝐓}_{𝐀},s)$ takes as input two matrices $𝐀\in {\mathbb{Z}}_{q}^{n\times m}$ and $𝐁\in {\mathbb{Z}}_{q}^{n\times m}$, a trapdoor ${𝐓}_{𝐀}$ for the lattice $\Lambda_{q}^{⟂}(𝐀)$, and a parameter $s\geq \|\tilde{{𝐓}_{𝐀}}\|\cdot \omega (\sqrt{\log m})$. The algorithm $\text{SampleBasisLeft}(𝐀,𝐁,{𝐓}_{𝐀},s)$ returns a basis ${𝐓}_{\left[𝐀|𝐁\right]}$ of $\Lambda_{q}^{⟂}([𝐀|𝐁])$ such that $\left[𝐀|𝐁]\cdot {𝐓}_{\left[𝐀|𝐁\right]}=0\;(\mathrm{mod}\;q\right)$.

For the polynomial-time algorithm $SampleBasisRight(𝐀,𝐆,𝐑,{𝐓}_{𝐆},s)$, it takes as input matrices $𝐀\in {\mathbb{Z}}_{q}^{n\times m}$, $𝐆\in {\mathbb{Z}}_{q}^{n\times m}$, a small-norm matrix $𝐑\in {\mathbb{Z}}^{m\times m}$, the trapdoor ${𝐓}_{𝐆}$ corresponding to the lattice $\Lambda_{q}^{⟂}(𝐆)$, along with a parameter *s* satisfying $s\geq \sqrt{5}(\|𝐑{\|}_{2}+1)\omega (\sqrt{\log m})$. The algorithm $\text{SampleBasisRight}(𝐀,𝐆,𝐑,{𝐓}_{𝐆},s)$ returns a basis ${𝐓}_{\left[𝐀|𝐀𝐑+𝐆\right]}$ for $\Lambda_{q}^{⟂}([𝐀|𝐀𝐑+𝐆])$ with the property that $\left[𝐀|𝐀𝐑+𝐆]\cdot {𝐓}_{\left[𝐀|𝐀𝐑+𝐆\right]}=0\;(\mathrm{mod}\;q\right)$.

For the polynomial-time algorithm $SampleLeft(𝐀,𝐁,{𝐓}_{𝐀},𝐃,s)$, with matrices $𝐀,𝐃\in {\mathbb{Z}}_{q}^{n\times m}$, $𝐁\in {\mathbb{Z}}_{q}^{n\times m_{1}}$, a short basis ${𝐓}_{𝐀}$ associated with the lattice $\Lambda_{q}^{⟂}(𝐀)$, and a parameter *s* chosen such that $s\geq \|\tilde{{𝐓}_{𝐀}}\|\cdot \omega (\sqrt{\log(m+m_{1})})$, it returns a matrix $𝐒\in {\mathbb{Z}}^{(m+m_{1})\times m}$ whose distribution is statistically close to $D_{\Lambda_{q}^{𝐃}({𝐅}_{1}),s}$, where ${𝐅}_{1}=(𝐀|𝐁)$.

For the polynomial-time algorithm $SampleRight(𝐀,𝐆,𝐒,{𝐓}_{𝐆},𝐃,s)$, it takes as input matrices $𝐀,𝐆\in {\mathbb{Z}}_{q}^{n\times m}$, a low-norm matrix $𝐒\in {\mathbb{Z}}_{q}^{m\times m}$, a short basis ${𝐓}_{𝐆}$ for the lattice $\Lambda_{q}^{⟂}(𝐆)$, a matrix $𝐃\in {\mathbb{Z}}_{q}^{n\times m_{1}}$, and a parameter $s\geq \sqrt{5}(\|𝐒{\|}_{2}+1)\omega (\sqrt{\log m})$. Running $SampleRight(𝐀,𝐆,𝐒,{𝐓}_{𝐆},𝐃,s)$ outputs a matrix $𝐑\in {\mathbb{Z}}^{2m\times m_{1}}$ whose distribution is statistically close to $D_{\Lambda_{q}^{𝐃}({𝐅}_{2}),s}$, where ${𝐅}_{2}=(𝐀|𝐀𝐒+𝐆)$.

Here, we introduce a variant of the SampleRight algorithm, denoted as SampleRightExtend, that supports the security analysis of the proposed framework. The algorithm is described as follows:

**Lemma 5.** Take integers n≥1, q≥2, and $\tilde{m}\geq n$. Set the parameter *s* such that $s\geq \sqrt{5}\;\|{𝐒}_{1}+{𝐒}_{2}{\|}_{2}+1\cdot \omega (\sqrt{\log(3m)})$. With matrices $𝐀,𝐆\in {\mathbb{Z}}_{q}^{n\times m}$, two matrices ${𝐒}_{1},{𝐒}_{2}\in \{0,1{\}}^{m\times m}$ of small norm, a short basis ${𝐓}_{𝐆}$ of the lattice $\Lambda_{q}^{⟂}(𝐆)$, and a uniformly chosen $𝐃\in {\mathbb{Z}}_{q}^{n\times \tilde{m}}$, a probabilistic polynomial-time algorithm $\text{SampleRightExtend}(𝐀,𝐆,{𝐒}_{1}+{𝐒}_{2},{𝐓}_{𝐆},𝐃,s)$ exists. This algorithm produces a matrix $𝐄\in {\mathbb{Z}}^{3m\times \tilde{m}}$ whose distribution is statistically indistinguishable from $D_{\Lambda_{q}^{D}(𝐅),s}$, where $𝐅=(𝐀|{𝐀}_{1}|{𝐀}_{2})$, with ${𝐀}_{i}=𝐀{𝐒}_{i}+r_{i}𝐆$, $r_{i}\in {\mathbb{Z}}_{q}^{n\times n}$ for i∈{1,2} and $r_{i}\in \{0,1\}$ is an invertible tag matrix.

### 2.4. Full-Rank Difference (FRD) Encoding [39]

In this scheme, we employ a Full-Rank Difference (FRD) function to encode user identities: $H:{\mathbb{Z}}_{q}^{n}⟶{\mathbb{Z}}_{q}^{n\times n}$. Suppose *n* > 1 and that *q* is an odd prime with q≥2. The FRD function is defined to satisfy the conditions below:

1. For any two distinct elements ${𝐱}_{1},{𝐱}_{2}\in {\mathbb{Z}}_{q}^{n}$ where ${𝐱}_{1}\text{≠}{𝐱}_{2}$, the difference matrix $H({𝐱}_{1})-H({𝐱}_{2})\in {\mathbb{Z}}_{q}^{n\times n}$ has full rank;
2. When the input is the zero vector $0\in {\mathbb{Z}}_{q}^{n}$, the FRD function outputs $H(0)=0\in {\mathbb{Z}}_{q}^{n\times n}$.
3. *H* is computable in polynomial time.

### 2.5. Complete subtree

In this construction, a complete binary tree structure [42] is employed to enhance efficiency. Its main purpose is to reduce the computational cost of handling ciphertexts for non-revoked users from linear complexity into a logarithmic scale. Specifically, let BT be a binary tree in which each leaf node is associated with a user identity id, and let root denote the root node of BT. We define Path(BT,id) as the sequence of nodes along the path from the leaf linked to *id* upward to the root. For any internal node *y*, let $y_{l}$ and $y_{r}$ denote its left and right child nodes. We denote by RL the revocation list, which maintains the identifiers of users subject to revocation. Based on this structure, KUNodes(BT,RL) is used to compute a minimal covering set Y that represents all non-revoked users. The procedure of KUNodes(BT,RL) is described in Table 3.

**Table 3:** KUNodes Algorithm.

Algorithm 1	KUNodes
**Input:**	*BT*, *RL*
**Output:**	*Y*
1	Set X=∅, Y=∅.
2	For each id∈RL do
3	Insert all nodes along Path(*BT*, *id*) into *X*.
4	End for
5	For each node y∈X do
6	If $y_{l}$ is not in *X* then
7	$Y=Y\cup \{y_{l}\}$.
8	End if
9	If $y_{r}$ is not in *X* then
10	$Y=Y\cup \{y_{r}\}$.
11	End if
12	End for
13	If *Y* is empty, then
14	*Y* = {root}.
15	End if
16	Return *Y*.

**Fig 1:**
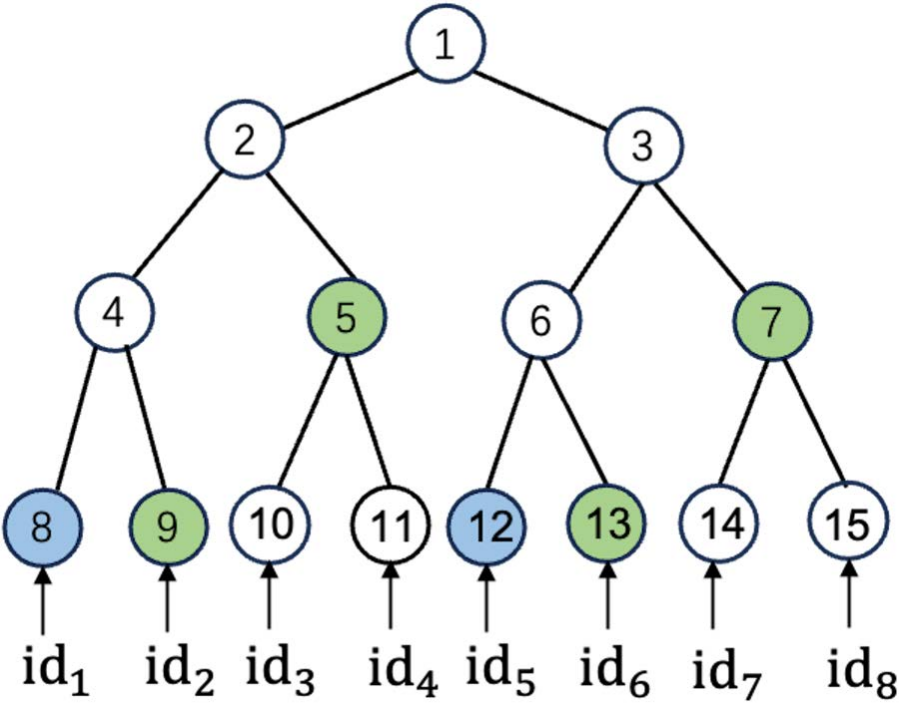
Binary Tree Example.

The operation of the KUNodes(*BT*, *RL*) algorithm is exemplified in Fig 1. Suppose that users *id*_1_ and *id*_5_ are revoked, i.e., $RL=\{id_{1},id_{5}\}=\{8,12\}$. According to the KUNodes(*BT*, *RL*) algorithm, the minimal covering set is *Y* = {5, 7, 9, 13}. For a non-revoked user *id*_8_, whose corresponding leaf node is 15, we have Path(*BT*, *id*_8_) = Path(15) = {1, 3, 7, 15}. Then $\text{Path}(id_{8})\cap \text{KUNodes}(BT,RL)=\{7\}$, which allows the non-revoked user to identify a valid covering node for decryption.

## 3. System definition

### 3.1. System model

**Fig 2:**
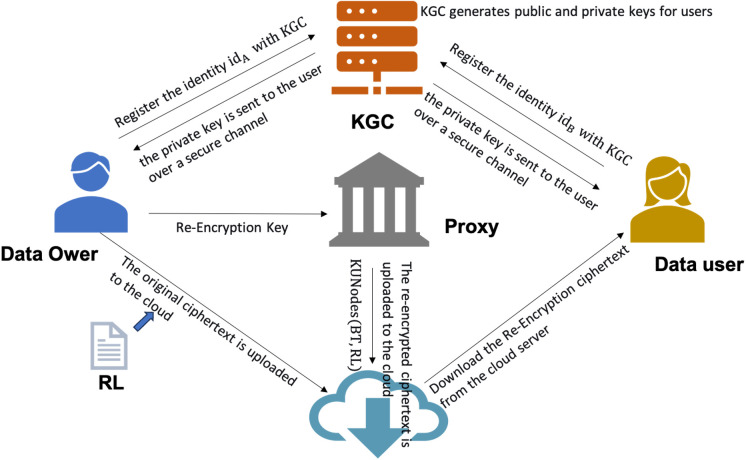
System Model of IBPRE-DR.

Fig 2 presents the system framework scheme. We define the system to include five types of entities, described as follows: KGC, Data Owner (User A), Proxy, Data User (User B), and Cloud Server.

- KGC: Initializes the system framework and generates the master public/private key pair. It generates partial private keys for all users (including data owners, data users, and the proxy) based on their identities. After the user registration phase, the KGC does not participate in daily data sharing and re-encryption processes.
- Data Owner: The data owner, such as a company manager or department director, is considered fully trusted. When a staff member joins or leaves the organization, the data owner updates access permissions by revoking the corresponding user. The data owner encrypts the data before uploading it to the cloud server.
- Proxy: The initial ciphertext, which is generated using the public key of the data owner, is obtained from the cloud server. The proxy retrieves the original ciphertext from the cloud server, uses the re-encryption key to transform it into a new ciphertext, and uploads the transformed ciphertext back to the cloud server. The authorized data user can then decrypt it with the corresponding private key.
- Data User: The data user, such as a company employee, downloads the ciphertext from the cloud server and decrypts it with the private key to recover the plaintext.
- Cloud Server: The cloud server provides data storage and retrieval services. It stores both the original ciphertext uploaded by the data owner and the transformed ciphertext generated by the proxy, responds to users’ requests, and returns the corresponding ciphertext.

The proxy and the cloud server are modeled as semi-trusted honest-but-curious entities. They are assumed to follow the prescribed protocol honestly, while possibly attempting to infer sensitive information from the ciphertexts, re-encryption keys, and intermediate values available to them.

### 3.2. Identity-based direct revocation proxy re-encryption scheme

We define the IBPRE-DR construction, which includes seven algorithms described as follows: Setup, KeyGen, Encrypt, ReKeyGen, ReEncrypt, Decrypt, and ReDecrypt. Each algorithm is specified below:

**Setup**$\left(1^{\lambda })\to (MPK,MSK\right)$: A semi-trusted authority executes this algorithm. The algorithm receives the security parameter λ and outputs the master public key MPK, the master secret key MSK, and a state list ST.

**KeyGen**$(MSK,id)\to {sk}_{id}$: Taking the master secret key MSK and a user identity id as input, it produces the corresponding private key ${sk}_{id}$.

**Encrypt**$(MPK,{id}_{A},\mu ,RL)\to C_{{id}_{A}}$: On input the master public key MPK, the identity ${id}_{A}$ belonging to user A, a message μ, and the revocation list RL, the algorithm outputs a ciphertext $C_{{id}_{A}}$.

**ReKeyGen**$({sk}_{{id}_{A}},{id}_{A},{id}_{B},ST)\to {rk}_{A\to B,y}$: On input the private key ${sk}_{{id}_{A}}$ of user A, the identities ${id}_{A}$ and ${id}_{B}$ of users A and B, respectively, and the state list ST, the algorithm outputs a re-encryption key ${rk}_{A\to B,y}$, where $y\in Path({id}_{B})$. If ${id}_{A}\notin ST$, the state list is updated as $ST\leftarrow ST\cup \{{id}_{A}\}$.

**ReEncrypt**$(MPK,C_{{id}_{A}},{rk}_{A\to B,y})\to C_{{id}_{B}}$: On input the master public key MPK, the ciphertext $C_{{id}_{A}}$, and the re-encryption key ${rk}_{A\to B,y}$, the algorithm outputs a re-encrypted ciphertext $C_{{id}_{B}}=(C_{1},C_{y,\hat{y}})$, where $y\in Path({id}_{B})$ and $\hat{y}\in KUNodes(BT,RL)$.

**Decrypt**$({id}_{A},{sk}_{{id}_{A}},C_{{id}_{A}})\to \mu$: On input the identity ${id}_{A}$, the decryption key ${sk}_{{id}_{A}}$, and the original ciphertext $C_{{id}_{A}}$, the algorithm outputs the message μ.

**ReDecrypt**$({id}_{B},{sk}_{{id}_{B}},C_{{id}_{B}})\to \mu$: On input the identity ${id}_{B}$, the decryption key ${sk}_{{id}_{B}}$, and the re-encrypted ciphertext $C_{{id}_{B}}$, the algorithm outputs the message μ.

**Correctness**: A revocable proxy re-encryption scheme satisfies correctness if it fulfills the properties outlined below. With MPK produced by $Setup(1^{\lambda })$ and ${sk}_{id}\leftarrow KeyGen(MSK,id)$, there exists a negligible function negl(·) such that:

- **1. Correctness of original ciphertext decryption:** For any message μ and any ciphertext $C_{{id}_{A}}\leftarrow Encrypt(MPK,{id}_{A},\mu ,RL)$, we have
  $$\begin{array}{c} \mathrm{Pr}[Decrypt({id}_{A},{sk}_{{id}_{A}},C_{{id}_{A}})=\mu ]>1-negl(\lambda ). \end{array}$$
- **2. Correctness of re-encrypted ciphertext decryption:** For any message μ, if $C_{{id}_{B}}\leftarrow ReEncrypt(MPK,C_{{id}_{A}},{rk}_{A\to B,y})$ is a re-encrypted ciphertext, where
  $$\begin{array}{c} {rk}_{A\to B,y}\leftarrow ReKeyGen({sk}_{{id}_{A}},{id}_{A},{id}_{B},ST) \end{array}$$
  and
  $$\begin{array}{c} C_{{id}_{A}}\leftarrow Encrypt(MPK,{id}_{A},\mu ,RL), \end{array}$$
  then we have
  $$\begin{array}{c} \mathrm{Pr}[ReDecrypt({id}_{B},{sk}_{{id}_{B}},C_{{id}_{B}})=\mu ]>1-negl(\lambda ). \end{array}$$

### 3.3. Security model

We define selective-identity chosen-plaintext security (IND-sID-CPA) for the IBPRE-DR using the following game between a challenger 𝒞 and an adversary 𝒜.

**Init:** 𝒜 challenge identity ${id}^{*}$ and a revocation list ${RL}^{*}$ are announced by the adversary 𝒜.

**Setup:** Upon executing $Setup(1^{\lambda })$, the challenger 𝒞 produces the master public key MPK alongside the master secret key MSK. The public parameters MPK are then provided to 𝒜, while MSK is retained as confidential.

**Phase 1:** Throughout the game, 𝒜 may adaptively issue polynomially many queries of the following forms:

**Private key query** ${𝒪}_{KeyGen}$: 𝒜 issues a private key query for an identity id where $id\text{≠}{id}^{*}$; alternatively the challenger returns ⊥. 𝒞 executes KeyGen(MSK,id) and returns ${sk}_{id}$ to 𝒜.

**Re-encryption key query** ${𝒪}_{ReKeyGen}$: 𝒜 submits a pair $\left({id}_{A},{id}_{B}\right)$. If ${id}_{A}\in ST$ and ${id}_{A}\text{≠}{id}^{*}$, the challenger returns ⊥. Otherwise, 𝒞 runs $ReKeyGen({sk}_{{id}_{A}},{id}_{A},{id}_{B},ST)$, returns the re-encryption key ${rk}_{A\to B,y}$, and updates the state list ST.

**Re-encryption query** ${𝒪}_{ReEncrypt}$: 𝒜 submits an identity pair and a ciphertext. If the re-encryption key already exists (or if ${id}_{A}\in ST$), the challenger directly executes ReEncrypt and returns the result. Otherwise, if ${id}_{A}\notin ST$, the challenger first generates the re-encryption key via ReKeyGen, updates ST, then performs $ReEncrypt(MPK,C_{{id}_{A}},{rk}_{A\to B,y})$ and returns the re-encrypted ciphertext $C_{{id}_{B}}$.

**Challenge**: 𝒜 chooses two messages *μ*_0_ and *μ*_1_ of identical length and submits them, along with the designated target identity ${id}^{*}$ during the initialization stage. The challenger 𝒞 then selects a random bit b∈{0,1} and generates the challenge ciphertext $C^{*}\leftarrow Encrypt(MPK,{id}^{*},\mu_{b},{RL}^{*})$. Subsequently, $C^{*}$ is returned to 𝒜.

**Phase 2**: The adversary continues to issue queries as in Phase 1 under the same constraints.

**Guess**: 𝒜 produces a bit $b^{*}\in \{0,1\}$ as its guess. If $b^{*}=b$, 𝒜 is considered successful and the experiment outputs 1; otherwise, the output is 0.

The adversary 𝒜’s advantage in the aforementioned game is given by


$$\begin{array}{c} {Adv}_{𝒜}^{IND\text{-}sID\text{-}CPA}(1^{\lambda })=\left|\mathrm{Pr}[b^{*}=b]-\frac{1}{2}\right| \end{array}$$

**Definition 5.** In the standard model, the presented IBPRE-DR scheme is considered IND-sID-CPA secure if, for any adversary 𝒜 performing in probabilistic polynomial time, the associated advantage ${Adv}_{𝒜}^{CPA}(1^{\lambda })$ is a negligible function of the security parameter λ.

## 4. IBPRE-DR Scheme

The IBPRE-DR scheme consists of the following seven algorithms.

**Setup**$\left(1^{\lambda },N\right)$:

1. Input a security parameter λ and the maximum number of users *N*. Run TrapGen(λ,m,q) to obtain a matrix ${𝐀}_{0}\in {\mathbb{Z}}_{q}^{n\times m}$ and its associated trapdoor ${𝐓}_{{𝐀}_{0}}\in {\mathbb{Z}}^{m\times m}$. The generated trapdoor satisfies $\left\|\tilde{{𝐓}_{{𝐀}_{0}}}\|\leq O(\sqrt{n\log q}\right)$, and σ is subsequently set such that $\sigma \geq \|\tilde{{𝐓}_{{𝐀}_{0}}}\|\cdot \omega (\sqrt{\log m})$.
2. Randomly choose two matrices ${𝐀}_{1}\in {\mathbb{Z}}_{q}^{n\times m}$ and $𝐃\in {\mathbb{Z}}_{q}^{n\times \ell }$.
3. Construct a complete binary tree BT containing *N* leaf nodes. For any node y∈BT, randomly select a uniformly distributed matrix ${𝐔}_{y}\in {\mathbb{Z}}_{q}^{n\times m}$. Set the initial state list ST:=BT.
4. The master public key is defined as $MPK=({𝐀}_{0},{𝐀}_{1},𝐃,BT,H)$, while the master secret key is defined as $MSK={𝐓}_{{𝐀}_{0}}$.

**KeyGen**(MSK,id):

1. Input the master secret key MSK and a user identity id.
2. Encode the identity as ${𝐅}_{id}=({𝐀}_{0}|{𝐀}_{1}+H(id)𝐆)\in {\mathbb{Z}}_{q}^{n\times 2m}$. Denote $𝐁={𝐀}_{1}+H(id)𝐆$.
3. Run $SampleBasisLeft({𝐀}_{0},𝐁,{𝐓}_{{𝐀}_{0}},s)$ to derive a basis ${𝐒}_{id}$ for the lattice $\Lambda_{q}^{⟂}({𝐅}_{id})$ satisfying ${𝐅}_{id}\cdot {𝐒}_{id}=0\;(\mathrm{mod}\;q)$.
4. Run $SamplePre({𝐅}_{id},{𝐒}_{id},𝐃,s)$ to obtain a matrix ${𝐄}_{id}\in {\mathbb{Z}}_{q}^{2m\times l}$ such that ${𝐅}_{id}\cdot {𝐄}_{id}=𝐃\;(\mathrm{mod}\;q)$.
5. Output the private key as ${sk}_{id}=({𝐒}_{id},{𝐄}_{id})$.

**Encrypt**$\left(MPK,{id}_{A},\mu ,RL\right)$:

1. Input the master public key MPK, the identity ${id}_{A}$ of user A, a message $\mu \in \{0,1{\}}^{\ell }$, and the revocation list RL.
2. Select a uniformly random vector $𝐬\in {\mathbb{Z}}_{q}^{n}$, noises ${𝐱}_{0}\in \psi^{m}$, ${𝐱}_{1}\in \psi^{\ell }$, and for $\hat{y}\in KUNodes(BT,RL)$, randomly select two matrices ${𝐒}_{1},{𝐒}_{\hat{y}}\in \{-1,1{\}}^{m\times m}$.
3. Let ${𝐅}_{{id}_{A}}=({𝐀}_{0}|{𝐀}_{1}+H({id}_{A})𝐆)\in {\mathbb{Z}}_{q}^{n\times 2m}$, and define $𝐱=({𝐈}_{m},{𝐒}_{1}{)}^{⊤}{𝐱}_{0}\in {\mathbb{Z}}_{q}^{2m}$, where ${𝐈}_{m}$ is the identity matrix.
4. Compute the ciphertext components:
  $$\begin{array}{c} {𝐂}_{0}={𝐅}_{{id}_{A}}^{⊤}𝐬+𝐱\in {\mathbb{Z}}_{q}^{2m}; \end{array}$$
  $$\begin{array}{c} {𝐂}_{1}={𝐃}^{⊤}𝐬+{𝐱}_{1}+\mu \left\lfloor q/2\right\rfloor \in {\mathbb{Z}}_{q}^{\ell }; \end{array}$$
  $$\begin{array}{c} {𝐂}_{\hat{y}}={𝐔}_{\hat{y}}^{⊤}𝐬+{𝐒}_{\hat{y}}^{⊤}{𝐱}_{0}\in {\mathbb{Z}}_{q}^{m}, \end{array}$$
  where $\hat{y}\in KUNodes(BT,RL)$.
5. Output the ciphertext ${𝐂}_{{id}_{A}}=({𝐂}_{0},{𝐂}_{1},{𝐂}_{\hat{y}})$.

**ReKeyGen**$\left({sk}_{{id}_{A}},{id}_{A},{id}_{B},ST\right)$:

1. If ST already contains ${id}_{A}$, return ⊥; otherwise, insert ${id}_{A}$ into ST by setting $ST\leftarrow ST\cup \{{id}_{A}\}$.
2. Let ${𝐅}_{{id}_{A},y}=({𝐀}_{0}|{𝐀}_{1}+H({id}_{A})𝐆|{𝐔}_{y})$, and ${𝐅}_{{id}_{B}}=({𝐀}_{0}|{𝐀}_{1}+H({id}_{B})𝐆)$.
3. For $y\in Path({id}_{B})$, run $SampleLeft({𝐅}_{{id}_{A}},{𝐒}_{{id}_{A}},{𝐅}_{{id}_{B}},{𝐔}_{y},s)\to {𝐑}_{y}$, satisfying ${𝐅}_{{id}_{A},y}\cdot {𝐑}_{y}={𝐅}_{{id}_{B}}$, where ${𝐑}_{y}\in {\mathbb{Z}}_{q}^{3m\times 2m}$.
4. Output the re-encryption key ${rk}_{A\to B,y}={𝐑}_{y}$, where $y\in Path({id}_{B})$, and update the state ST.

**ReEncrypt**$\left(MPK,{𝐂}_{{id}_{A}},{𝐑}_{y}\right)$:

1. Input user A’s ciphertext ${𝐂}_{{id}_{A}}=({𝐂}_{0},{𝐂}_{1},{𝐂}_{\hat{y}})$, master public key MPK, and re-encryption key ${𝐑}_{y}$.
2. Compute ${𝐂}_{y,\hat{y}}={𝐑}_{y}^{⊤}({𝐂}_{0}|{𝐂}_{\hat{y}})\in {\mathbb{Z}}_{q}^{2m}$, where $y\in Path({id}_{B})$ and $\hat{y}\in KUNodes(BT,RL)$.
3. Output the re-encrypted ciphertext ${𝐂}_{{id}_{B}}=({𝐂}_{1},{𝐂}_{y,\hat{y}})$, where $y\in Path({id}_{B})$ and $\hat{y}\in KUNodes(BT,RL)$.

**Decrypt**$\left({id}_{A},{sk}_{{id}_{A}},{𝐂}_{{id}_{A}}\right)$:

1. Input the identity ${id}_{A}$, the private key ${sk}_{{id}_{A}}=({𝐒}_{{id}_{A}},{𝐄}_{{id}_{A}})$, and the ciphertext ${𝐂}_{{id}_{A}}=({𝐂}_{0},{𝐂}_{1},{𝐂}_{\hat{y}})$.
2. Compute the intermediate vector:
  $$\begin{array}{c} 𝐌={𝐂}_{1}-{𝐄}_{{id}_{A}}^{⊤}{𝐂}_{0}. \end{array}$$
3. Recover each message bit $\mu_{i}$ for i=1,...,ℓ as:
  $$\begin{array}{c} \mu_{i}=\left\{ \begin{array}{cc} 1, & \text{if }\left|M_{i}-\left\lfloor \frac{q}{2}\right\rfloor \right|<\frac{q}{4},\\ 0, & \text{otherwise}, \end{array}\right. \end{array}$$
  where $M_{i}$ denotes the *i*-th component of 𝐌.
4. Output the message vector $\mu =(\mu_{1},...,\mu_{\ell })\in \{0,1{\}}^{\ell }$.

**ReDecrypt**$\left({id}_{B},{sk}_{{id}_{B}},{𝐂}_{{id}_{B}}\right)$:

1. Input the identity ${id}_{B}$, the private key ${sk}_{{id}_{B}}=({𝐒}_{{id}_{B}},{𝐄}_{{id}_{B}})$, and the re-encrypted ciphertext ${𝐂}_{{id}_{B}}=({𝐂}_{1},\{{𝐂}_{y,\hat{y}}{\}}_{y\in Path({id}_{B}),\;\hat{y}\in KUNodes(BT,RL)})$.
2. For each valid pair $\left(y,\hat{y}\right)$, compute the intermediate vector:
  $$\begin{array}{c} {𝐌}_{y,\hat{y}}={𝐂}_{1}-{𝐄}_{{id}_{B}}^{⊤}{𝐂}_{y,\hat{y}}. \end{array}$$
3. Recover each message bit $\mu_{i}$ for i=1,...,ℓ as:
  $$\begin{array}{c} \mu_{i}=\left\{ \begin{array}{cc} 1, & \text{if }\left|M_{y,\hat{y},i}-\left\lfloor \frac{q}{2}\right\rfloor \right|<\frac{q}{4},\\ 0, & \text{otherwise}, \end{array}\right. \end{array}$$
  where $M_{y,\hat{y},i}$ denotes the *i*-th component of ${𝐌}_{y,\hat{y}}$.
4. If there exists a valid pair $\left(y,\hat{y}\right)$ such that the above condition holds for all *i*, output the message vector $\mu =(\mu_{1},...,\mu_{\ell })\in \{0,1{\}}^{\ell }$; otherwise, output ⊥.

**Fig 3:**
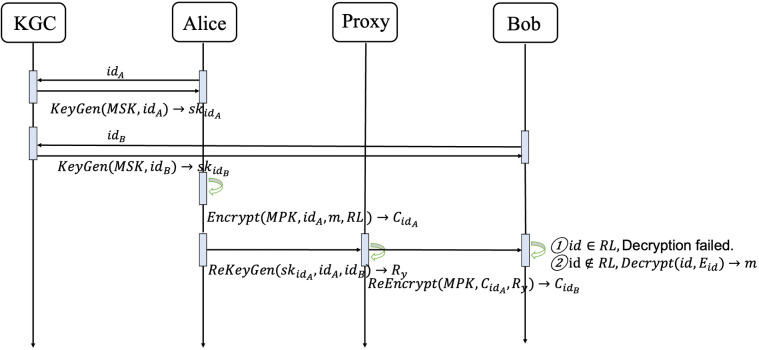
Algorithmic Interplay Procedure.

We illustrate the workflow of the proposed IBPRE-DR scheme in Fig 3. The Key Generation Center (KGC) serves as the central authority responsible for system initialization and private key generation. Alice and Bob first register their identities with the KGC. Based on the registered identities, the KGC generates the corresponding private keys and securely distributes them to the respective users.

Alice encrypts the message under her identity and sends the resulting ciphertext to the proxy through a secure communication channel. The proxy then uses the corresponding re-encryption key to transform Alice’s ciphertext into a ciphertext intended for Bob, without learning any information about the underlying plaintext.

During decryption, the revocation status of Bob is checked according to the revocation list. If Bob’s identity is included in the revocation list, he is not allowed to decrypt the ciphertext. Otherwise, Bob uses his private decryption key to recover the original plaintext from the re-encrypted ciphertext.

### 4.1. Parameters and correctness

#### 4.1.1. Parameters.

The following parameter settings are adopted to ensure the scheme’s correctness and security:

(1) **Security and dimension parameters:**
  - Security parameter λ=n;
  - Maximum number of users *N*;
  - Identity encoding dimension l=poly(n);
  - Message encoding dimension ℓ=ω(logn);
  - Number of columns in matrices $m=\left\lceil 2n\left\lceil \log_{2}q\right\rceil \right\rceil$;
  - Noise bound condition: *q*/*B* > 4(*m* + 1)^8^, where *B* is the noise bound.
(2) **Gaussian parameters:**
  - For key generation algorithms SampleBasisRight and SampleBasisLeft: $s=\omega ((m+1{)}^{2})$;
  - For decryption algorithm SamplePre: $\sigma_{1}=\omega ((m+1{)}^{2}\sqrt{\log(2m)})$, ensuring noise remains manageable;
  - For re-encryption key generation procedures SampleLeft and SampleRightExtend: $\sigma_{2}=\omega ((m+1{)}^{5/2}\sqrt{\log(3m)})$, to account for noise accumulation during encryption.

Following the concrete parameter selection method used in several LWE-based schemes, we instantiate the underlying LWE parameters for practical performance evaluation as follows. To target an estimated 128-bit post-quantum security level, we set *n* = 1024, ℓ=256, and choose a 27-bit prime modulus *q*.

#### 4.1.2. Correctness.

**Decryption of the original ciphertext.** Let ${sk}_{{id}_{A}}:=({𝐒}_{{id}_{A}},{𝐄}_{{id}_{A}})$ be the private key of user *A*. For the original ciphertext ${𝐂}_{{id}_{A}}=({𝐂}_{0},{𝐂}_{1},{𝐂}_{\hat{y}})$, the decoding procedure only uses the components ${𝐂}_{0}$ and ${𝐂}_{1}$. Since ${𝐅}_{{id}_{A}}{𝐄}_{{id}_{A}}=𝐃\;(\mathrm{mod}\;q)$ and $\|{𝐄}_{{id}_{A}}\|\leq \sigma_{1}\sqrt{2m}\ell$ hold with overwhelming probability, we have


$$\begin{array}{cc} {𝐂}_{1}-{𝐄}_{{id}_{A}}^{⊤}{𝐂}_{0} & ={𝐂}_{1}-{𝐄}_{{id}_{A}}^{⊤}\left({𝐅}_{{id}_{A}}^{⊤}𝐬+𝐱\right)\\ & ={𝐃}^{⊤}𝐬+{𝐱}_{1}+\left\lfloor \frac{q}{2}\right\rfloor \mu -\left({𝐄}_{{id}_{A}}^{⊤}{𝐅}_{{id}_{A}}^{⊤}𝐬+{𝐄}_{{id}_{A}}^{⊤}𝐱\right)\\ & ={𝐃}^{⊤}𝐬+{𝐱}_{1}+\left\lfloor \frac{q}{2}\right\rfloor \mu -\left({𝐃}^{⊤}𝐬+{𝐄}_{{id}_{A}}^{⊤}𝐱\right)\\ & =\left\lfloor \frac{q}{2}\right\rfloor \mu +\underset{\text{noise}}{\underbrace{\left({𝐱}_{1}-{𝐄}_{{id}_{A}}^{⊤}𝐱\right)}}. \end{array}$$

The noise term satisfies


$$\begin{array}{c} {\left\|{𝐱}_{1}-{𝐄}_{{id}_{A}}^{⊤}𝐱\right\|}_{\infty }\leq B\sqrt{\ell }+(\sigma_{1}\sqrt{2m}\ell )(21Bm)\leq B(m+1{)}^{5}<\frac{q}{4}. \end{array}$$

Therefore, for each component *i*, we have $M_{i}=\left\lfloor q/2\right\rfloor \mu_{i}+{noise}_{i}$. Since $|{noise}_{i}|<q/4$, the value $M_{i}$ is closer to 0 when $\mu_{i}=0$, and closer to ⌊q/2⌋ when $\mu_{i}=1$. Hence, the threshold decoding algorithm correctly recovers each bit $\mu_{i}$, and consequently recovers the message vector μ with overwhelming probability.

**Re-encryption of the original ciphertext.** Let *y* be a valid node such that $y\in Path({id}_{B})\cap KUNodes(BT,RL)$. The corresponding re-encrypted ciphertext component is denoted by ${𝐂}_{y}$. Given the re-encryption key ${rk}_{A\to B,y}=\{{𝐑}_{y}\}$, where

$\left({𝐀}_{0}|{𝐀}_{1}+H({id}_{A})𝐆|{𝐔}_{y}\right){𝐑}_{y}=\left({𝐀}_{0}|{𝐀}_{1}+H({id}_{B})𝐆\right)\;(\mathrm{mod}\;q),$ and $\|{𝐑}_{y}\|\leq \sigma_{2}m\sqrt{6}$ with overwhelming probability, we have


$$\begin{array}{cc} {𝐂}_{y} & ={𝐑}_{y}^{⊤}[\begin{array}{c} {𝐂}_{0}\\ {𝐂}_{y}^{\prime } \end{array}]\\ & ={𝐑}_{y}^{⊤}\left({\left[{𝐅}_{{id}_{A}}|{𝐔}_{y}\right]}^{⊤}𝐬+[\begin{array}{c} 𝐱\\ {𝐒}_{y}^{⊤}{𝐱}_{0} \end{array}]\right)\\ & ={\left(\left[{𝐅}_{{id}_{A}}|{𝐔}_{y}\right]{𝐑}_{y}\right)}^{⊤}𝐬+{𝐑}_{y}^{⊤}[\begin{array}{c} 𝐱\\ {𝐒}_{y}^{⊤}{𝐱}_{0} \end{array}]\\ & ={𝐅}_{{id}_{B}}^{⊤}𝐬+\underset{noise}{\underbrace{{𝐑}_{y}^{⊤}[\begin{array}{c} 𝐱\\ {𝐒}_{y}^{⊤}{𝐱}_{0} \end{array}]}}. \end{array}$$

The noise term satisfies


$$\begin{array}{c} \left\|{𝐑}_{y}^{⊤}[\begin{array}{c} 𝐱\\ {𝐒}_{y}^{⊤}{𝐱}_{0} \end{array}]\right\|\leq \sigma_{2}m\sqrt{6}\cdot 30Bm. \end{array}$$

Thus, the re-encrypted ciphertext component has the same form as a ciphertext encrypted under ${id}_{B}$, except for a bounded noise term. Under the chosen parameter setting, this noise remains below the decoding threshold. Therefore, user *B* can correctly recover the message vector μ with overwhelming probability.

**Decryption of the re-encrypted ciphertext** $\left({𝐂}_{1},{𝐂}_{y,\hat{y}}\right)$. Let ${sk}_{{id}_{B}}:=({𝐒}_{{id}_{B}},{𝐄}_{{id}_{B}})$ be the private key of user *B*. Since ${𝐅}_{{id}_{B}}{𝐄}_{{id}_{B}}=𝐃\;(\mathrm{mod}\;q)$ and $\|{𝐄}_{{id}_{B}}\|\leq \sigma_{1}\sqrt{2m}\ell$ hold with overwhelming probability, we have


$$\begin{array}{cc} {𝐂}_{1}-{𝐄}_{{id}_{B}}^{⊤}{𝐂}_{y,\hat{y}} & ={𝐂}_{1}-{𝐄}_{{id}_{B}}^{⊤}\left({𝐅}_{{id}_{B}}^{⊤}𝐬+{𝐑}_{y}^{⊤}\left(𝐱+{𝐒}_{\hat{y}}^{⊤}{𝐱}_{0}\right)\right)\\ & ={𝐂}_{1}-{𝐃}^{⊤}𝐬-{𝐄}_{{id}_{B}}^{⊤}{𝐑}_{y}^{⊤}\left(𝐱+{𝐒}_{\hat{y}}^{⊤}{𝐱}_{0}\right)\\ & ={𝐃}^{⊤}𝐬+{𝐱}_{1}+\left\lfloor \frac{q}{2}\right\rfloor \mu -{𝐃}^{⊤}𝐬-{𝐄}_{{id}_{B}}^{⊤}{𝐑}_{y}^{⊤}\left(𝐱+{𝐒}_{\hat{y}}^{⊤}{𝐱}_{0}\right)\\ & =\left\lfloor \frac{q}{2}\right\rfloor \mu +\underset{noise}{\underbrace{\left({𝐱}_{1}-{𝐄}_{{id}_{B}}^{⊤}{𝐑}_{y}^{⊤}\left(𝐱+{𝐒}_{\hat{y}}^{⊤}{𝐱}_{0}\right)\right)}}. \end{array}$$

If the noise term is bounded by the decoding threshold, namely


$$\begin{array}{c} {\left\|{𝐱}_{1}-{𝐄}_{{id}_{B}}^{⊤}{𝐑}_{y}^{⊤}\left(𝐱+{𝐒}_{\hat{y}}^{⊤}{𝐱}_{0}\right)\right\|}_{\infty }<\frac{q}{4}, \end{array}$$

then each component can be correctly decoded as


$$\begin{array}{c} \mu_{i}=\left\{ \begin{array}{cc} 1, & \text{if }\left|M_{i}-\left\lfloor \frac{q}{2}\right\rfloor \right|<\frac{q}{4},\\ 0, & \text{otherwise}. \end{array}\right. \end{array}$$

Therefore, user *B* correctly recovers the message vector μ from the re-encrypted ciphertext with overwhelming probability.

To analyze revocation correctness, we consider the following two cases.

- **Case 1:** ${id}_{B}\notin RL$**.** If user *B* is not revoked, then there exists at least one node on $Path({id}_{B})$ that is also included in KUNodes(BT,RL). That is,
  $$\begin{array}{c} Path({id}_{B})\cap KUNodes(BT,RL)\text{≠}\emptyset . \end{array}$$

Let *y* be such a valid node and set $\hat{y}=y$. Then the corresponding ciphertext component can be used in the re-decryption algorithm. The resulting noise term is bounded as follows:


$$\begin{array}{cc} & {\left\|{𝐱}_{1}-{𝐄}_{{id}_{B}}^{⊤}{𝐑}_{y}^{⊤}\left(𝐱+{𝐒}_{\hat{y}}^{⊤}{𝐱}_{0}\right)\right\|}_{\infty }\\ & \;\leq B\sqrt{\ell }+\sigma_{1}\sqrt{2m}\ell \cdot \left(\sigma_{2}m\sqrt{6}\cdot 30Bm\right)\\ & \;\leq B(m+1)+B(m+1{)}^{8}\\ & \;\leq 2B(m+1{)}^{8}\leq \frac{q}{4}. \end{array}$$

Therefore, the noise remains below the decoding threshold. Hence, the threshold decoding algorithm correctly recovers each bit $\mu_{i}$, and consequently recovers the message vector μ with overwhelming probability.

- **Case 2:** ${id}_{B}\in RL$**.** If user *B* is revoked, then no node on $Path({id}_{B})$ is contained in KUNodes(BT,RL). Thus,
  $$\begin{array}{c} Path({id}_{B})\cap KUNodes(BT,RL)=\emptyset . \end{array}$$

In this case, no valid pair $\left(y,\hat{y}\right)$ can be found for the re-decryption procedure. Therefore, the re-decryption algorithm cannot obtain a valid ciphertext component for user *B*, and it outputs ⊥.

### 4.2. Security

**Theorem 4.1.** Assume that the decisional ${LWE}_{n,q,\chi }$ problem is hard for any probabilistic polynomial-time adversary. Then the proposed IBPRE-DR scheme is IND-sID-CPA secure in the standard model. More precisely, for any PPT adversary 𝒜 making polynomially many private-key, re-encryption-key, and re-encryption queries, there exists a PPT LWE distinguisher ℬ such that


$$\begin{array}{c} {Adv}_{𝒜}^{IND\text{-}sID\text{-}CPA}(1^{\lambda })\leq \left(|KUNodes(BT,{RL}^{*})|+2\right)\cdot {Adv}_{ℬ}^{LWE}(1^{\lambda })+negl(\lambda ). \end{array}$$

Since $|KUNodes(BT,{RL}^{*})|=O\left(r\log\frac{N}{r}\right)$, where $r=|{RL}^{*}|$, the reduction loss is linear in the number of revocation-related challenge components and remains polynomial in the security parameter.

**Proof.** We prove the theorem by a sequence of games. Let


$$\begin{array}{c} {Adv}_{i}(1^{\lambda })=\left|\mathrm{Pr}[{Game}_{i}=1]-\frac{1}{2}\right|, \end{array}$$

where ${Game}_{i}=1$ denotes the event that the adversary correctly guesses the challenge bit in ${Game}_{i}$.

**Query Restrictions.** Before presenting the games, we specify the legal query restrictions in the IND-sID-CPA experiment. In the selective-identity setting, the adversary 𝒜 first commits to a challenge identity ${id}^{*}$ and a challenge revocation list ${RL}^{*}$. The following restrictions are imposed to prevent trivial attacks:

- **Private-key queries:** 𝒜 is not allowed to query the private key of the challenge identity ${id}^{*}$.
- **Re-encryption-key queries:** The adversary is not allowed to query a re-encryption key from the challenge identity ${id}^{*}$ to any identity ${id}_{B}$ satisfying $Path({id}_{B})\cap KUNodes(BT,{RL}^{*})\text{≠}\emptyset$.
- **Re-encryption queries:** 𝒜 is not allowed to submit the challenge ciphertext to the re-encryption oracle in a way that produces a ciphertext decryptable by a non-revoked user.

All other queries are answered normally according to the corresponding algorithms.

**Game Sequence.** Through a sequence of four games, we show that the advantage of any PPT adversary 𝒜 is negligible.

- Game0 is the original IND-sID-CPA security game as defined in Definition 5.
- Game1 modifies the distribution of the public parameters using the Leftover Hash Lemma.
- Game2 replaces the trapdoor-based query responses with simulated responses using a public trapdoor.
- Game3 replaces the challenge ciphertext with a uniformly random element, yielding zero adversary advantage.

We prove that consecutive games are indistinguishable: Game0 and Game1 are statistically indistinguishable; Game1 and Game2 are statistically indistinguishable; and Game2 and Game3 are computationally indistinguishable under the LWE hardness assumption. Therefore, 𝒜’s advantage in Game0 is negligible.

- **Game0**. This is the real IND-sID-CPA security game defined in Section 3.3. The challenger runs the real setup algorithm to obtain (MPK,MSK), and answers all legal oracle queries honestly. In the challenge phase, the adversary submits two equal-length messages $\mu_{0},\mu_{1}\in \{0,1{\}}^{\ell }$. The challenger chooses a random bit b←{0,1} and returns the challenge ciphertext ${𝐂}^{*}\leftarrow Encrypt(MPK,{id}^{*},\mu_{b},{RL}^{*})$. By definition,
  $$\begin{array}{c} {Adv}_{0}={Adv}_{𝒜}^{IND\text{-}sID\text{-}CPA}(1^{\lambda }). \end{array}$$
- **Game1**. This game modifies the generation of some public matrices, while keeping the adversary’s view statistically close to that in Game0.

First, instead of choosing ${𝐀}_{1}$ uniformly at random, the challenger samples a small matrix ${𝐒}^{*}\leftarrow \{-1,1{\}}^{m\times m}$ and sets


$$\begin{array}{c} {𝐀}_{1}={𝐀}_{0}{𝐒}^{*}-H({id}^{*})𝐆. \end{array}$$

Therefore, for the challenge identity ${id}^{*}$, we have


$$\begin{array}{c} {𝐀}_{1}+H({id}^{*})𝐆={𝐀}_{0}{𝐒}^{*}. \end{array}$$

Next, for each node y∈BT, the challenger samples a small matrix ${𝐒}_{y}\leftarrow \{-1,1{\}}^{m\times m}$ and defines


$$\begin{array}{c} {𝐔}_{y}=\left\{ \begin{array}{cc} {𝐀}_{0}{𝐒}_{y}, & \text{if }y\in KUNodes(BT,{RL}^{*}),\\ {𝐀}_{0}{𝐒}_{y}-𝐆, & \text{otherwise}. \end{array}\right. \end{array}$$

All other public parameters are generated in the same way as in Game0.

By the leftover hash lemma, the distributions of ${𝐀}_{0}{𝐒}^{*}$ and ${𝐀}_{0}{𝐒}_{y}$ are statistically close to uniform over the corresponding matrix spaces. Hence, the public parameters generated in Game1 are statistically indistinguishable from those in Game0. It follows that


$$\begin{array}{c} \left|{Adv}_{0}-{Adv}_{1}\right|\leq negl(\lambda ). \end{array}$$

- **Game 2.** In this game, the challenger 𝒞 modifies the generation of ${𝐀}_{0}$. Instead of generating $\left({𝐀}_{0},{𝐓}_{{𝐀}_{0}}\right)$ by the trapdoor generation algorithm, 𝒞 chooses ${𝐀}_{0}\leftarrow {\mathbb{Z}}_{q}^{n\times m}$ uniformly at random and does not possess the trapdoor ${𝐓}_{{𝐀}_{0}}$. All other public parameters are generated as in ${Game}_{1}$. Since the distribution of the matrix output by TrapGen is statistically close to uniform over ${\mathbb{Z}}_{q}^{n\times m}$, and since ${𝐓}_{{𝐀}_{0}}$ is never given to the adversary, this modification is statistically hidden from 𝒜.

Although 𝒞 does not know ${𝐓}_{{𝐀}_{0}}$, it can still answer all admissible oracle queries by using the trapdoor ${𝐓}_{𝐆}$ of the gadget matrix 𝐆. The simulations of the oracle queries are described as follows.

- ${𝒪}_{KeyGen}$: For a private-key query on an identity $id\text{≠}{id}^{*}$, define $\Delta_{id}=H(id)-H({id}^{*})$. By the construction in ${Game}_{1}$, we have
  $$\begin{array}{c} {𝐅}_{id}=[{𝐀}_{0}|{𝐀}_{1}+H(id)𝐆]=[{𝐀}_{0}|{𝐀}_{0}{𝐒}^{*}+\Delta_{id}𝐆]. \end{array}$$

Since $id\text{≠}{id}^{*}$, the matrix $\Delta_{id}$ satisfies the full-rank difference property. Therefore, the challenger can use ${𝐓}_{𝐆}$ to derive a trapdoor for the right part of ${𝐅}_{id}$. Specifically, 𝒞 runs


$$\begin{array}{c} {𝐒}_{{𝐅}_{id}}\leftarrow SampleBasisRight({𝐀}_{0},\Delta_{id}𝐆,{𝐒}^{*},{𝐓}_{𝐆},s), \end{array}$$

which outputs a short basis ${𝐒}_{{𝐅}_{id}}$ for $\Lambda_{q}^{⟂}({𝐅}_{id})$. Then 𝒞 runs


$$\begin{array}{c} {𝐄}_{id}\leftarrow SamplePre({𝐅}_{id},{𝐒}_{{𝐅}_{id}},𝐃,\sigma_{1}), \end{array}$$

obtaining ${𝐄}_{id}$ such that ${𝐅}_{id}{𝐄}_{id}=𝐃\;(\mathrm{mod}\;q)$. Finally, 𝒞 returns the private key ${sk}_{id}=({𝐒}_{{𝐅}_{id}},{𝐄}_{id})$ to the adversary.

- ${𝒪}_{ReKeyGen}$: The challenger responds to every admissible re-encryption-key query from ${id}_{A}$ to ${id}_{B}$. For a node *y*, define the extended matrix ${𝐅}_{{id}_{A},y}=[{𝐀}_{0}|{𝐀}_{1}+H({id}_{A})𝐆|{𝐔}_{y}]\in {\mathbb{Z}}_{q}^{n\times 3m}$. The simulation is divided into the following two cases.
- **Case 1:** ${id}_{A}\text{≠}{id}^{*}$. In this case, ${𝐀}_{1}+H({id}_{A})𝐆={𝐀}_{0}{𝐒}^{*}+(H({id}_{A})-H({id}^{*}))𝐆$. Since $H({id}_{A})-H({id}^{*})$ satisfies the full-rank difference property, 𝒞 can use ${𝐓}_{𝐆}$ to sample a short matrix ${𝐑}_{y}$. It runs
  $$\begin{array}{c} {𝐑}_{y}\leftarrow SampleRightExtend({𝐀}_{0},𝐆,{𝐒}^{*}+{𝐒}_{y},{𝐓}_{𝐆},{𝐅}_{{id}_{B}},\sigma_{2}), \end{array}$$
  obtaining ${𝐑}_{y}\in {\mathbb{Z}}_{q}^{3m\times 2m}$ such that ${𝐅}_{{id}_{A},y}{𝐑}_{y}={𝐅}_{{id}_{B}}\;(\mathrm{mod}\;q)$. The challenger returns ${𝐑}_{y}$ as the re-encryption key.
- **Case 2:** ${id}_{A}={id}^{*}$ and ${id}_{B}$ is revoked with respect to ${RL}^{*}$. Since $Path({id}_{B})\cap KUNodes(BT,{RL}^{*})=\emptyset$, for every node $y\in Path({id}_{B})$, we have $y\notin KUNodes(BT,{RL}^{*})$. Therefore, by the construction in Game1, we have ${𝐔}_{y}={𝐀}_{0}{𝐒}_{y}-𝐆$. Moreover, ${𝐀}_{1}+H({id}^{*})𝐆={𝐀}_{0}{𝐒}^{*}$. Hence, the extended matrix for ${id}^{*}$ and node *y* is ${𝐅}_{{id}^{*},y}=[{𝐀}_{0}|{𝐀}_{0}{𝐒}^{*}|{𝐀}_{0}{𝐒}_{y}-𝐆]$. Although the identity component does not contain a gadget term, the node component contains −𝐆. Thus, 𝒞 can still use ${𝐓}_{𝐆}$ to sample the re-encryption key. Specifically, it runs
  $$\begin{array}{c} {𝐑}_{y}\leftarrow SampleRightExtend({𝐀}_{0},𝐆,{𝐒}^{*}+{𝐒}_{y},{𝐓}_{𝐆},{𝐅}_{{id}_{B}},\sigma_{2}), \end{array}$$
  obtaining ${𝐑}_{y}\in {\mathbb{Z}}_{q}^{3m\times 2m}$ such that ${𝐅}_{{id}^{*},y}{𝐑}_{y}={𝐅}_{{id}_{B}}\;(\mathrm{mod}\;q)$. The challenger returns ${𝐑}_{y}$ as the re-encryption key.
- ${𝒪}_{ReEncrypt}$: For an admissible re-encryption query, 𝒞 first obtains the corresponding simulated re-encryption key ${𝐑}_{y}$ as described in ${𝒪}_{ReKeyGen}$. Then it runs the public re-encryption algorithm ReEncrypt and returns the resulting re-encrypted ciphertext to the adversary.

Using the above simulation, the challenger can answer all admissible queries without knowing ${𝐓}_{{𝐀}_{0}}$. Moreover, by the standard properties of SampleBasisRight, SamplePre, and SampleRightExtend, the simulated private keys and re-encryption keys are statistically close to those generated in the real scheme. Therefore, the adversary’s view in Game2 is statistically indistinguishable from that in Game1, and we have


$$\begin{array}{c} |{Adv}_{1}-{Adv}_{2}|\leq negl(\lambda ). \end{array}$$

- **Game 3.** This game is identical to Game2, except that the challenge ciphertext is replaced with a uniformly random ciphertext of the same form. In Game2, the challenge ciphertext is
  $$\begin{array}{c} {𝐂}^{*}=({𝐂}_{0}^{*},{𝐂}_{1}^{*},\{{𝐂}_{\hat{y}}^{*}{\}}_{\hat{y}\in KUNodes(BT,{RL}^{*})}), \end{array}$$
  where
  $$\begin{array}{c} {𝐂}_{0}^{*}={𝐅}_{{id}^{*}}^{⊤}𝐬+𝐱,\;{𝐂}_{1}^{*}={𝐃}^{⊤}𝐬+{𝐱}_{1}+\left\lfloor \frac{q}{2}\right\rfloor \mu_{b}, \end{array}$$
  and, for each $\hat{y}\in KUNodes(BT,{RL}^{*})$,
  $$\begin{array}{c} {𝐂}_{\hat{y}}^{*}={𝐔}_{\hat{y}}^{⊤}𝐬+{𝐒}_{\hat{y}}^{⊤}{𝐱}_{0}. \end{array}$$

We transform Game2 into Game3 through a sequence of hybrid games defined over the underlying LWE samples used in the challenge ciphertext. In each hybrid, one underlying LWE-type sample is replaced by a uniformly random vector of the same dimension, and all ciphertext components derived from that sample are updated consistently. In particular, the identity-related component ${𝐅}_{{id}^{*}}^{⊤}𝐬+𝐱$, the message-masking component ${𝐃}^{⊤}𝐬+{𝐱}_{1}$, and the revocation-related components associated with $\hat{y}\in KUNodes(BT,{RL}^{*})$ are replaced through at most


$$\begin{array}{c} |KUNodes(BT,{RL}^{*})|+2 \end{array}$$

hybrid transitions. Each transition is computationally indistinguishable from the previous one under the decisional LWE assumption. The transformed error terms, such as ${𝐒}_{\hat{y}}^{⊤}{𝐱}_{0}$, remain bounded under the parameter settings and are treated as part of the corresponding LWE-type ciphertext components.

If any two consecutive hybrids can be distinguished with non-negligible advantage, then this distinguisher can be used to construct a PPT algorithm ℬ that distinguishes LWE samples from uniformly random samples.

Therefore, there exists a PPT LWE distinguisher ℬ such that


$$\begin{array}{c} |{Adv}_{2}-{Adv}_{3}|\leq \left(|KUNodes(BT,{RL}^{*})|+2\right)\cdot {Adv}_{ℬ}^{LWE}(1^{\lambda }). \end{array}$$

In Game3, the challenge ciphertext is uniformly random and independent of the challenge bit *b*. Hence, the adversary obtains no information about whether $\mu_{0}$ or $\mu_{1}$ was encrypted. Therefore,


$$\begin{array}{c} \mathrm{Pr}[b^{\prime }=b]=\frac{1}{2},\;{Adv}_{3}=0. \end{array}$$

Combining all game transitions, we obtain


$$\begin{array}{cc} {Adv}_{𝒜}^{IND\text{-}sID\text{-}CPA}(1^{\lambda }) & ={Adv}_{0}\\ & \leq |{Adv}_{0}-{Adv}_{1}|+|{Adv}_{1}-{Adv}_{2}|+|{Adv}_{2}-{Adv}_{3}|+{Adv}_{3}\\ & \leq \left(|KUNodes(BT,{RL}^{*})|+2\right)\cdot {Adv}_{ℬ}^{LWE}(1^{\lambda })+negl(\lambda ). \end{array}$$

Since the decisional LWE problem is assumed to be hard, the advantage of ℬ is negligible. Therefore,


$$\begin{array}{c} {Adv}_{𝒜}^{IND\text{-}sID\text{-}CPA}(1^{\lambda }) \end{array}$$

is negligible. This proves that the proposed IBPRE-DR scheme is IND-sID-CPA secure in the standard model.

### 4.3. Comparison

The proposed IBPRE-DR scheme is compared with representative related schemes from several aspects, including security properties, functional features, theoretical complexity, computational overhead, estimated key and ciphertext sizes, and experimental running time. Specifically, Tables 4 and 5 summarize the security and functionality comparisons, Table 6 presents the theoretical comparison of identity-based revocable schemes, Table 7 compares the computational overhead of revocable proxy re-encryption schemes, Table 8 reports the estimated key and ciphertext sizes under the experimental parameters, and Table 9 gives the measured running time of the main algorithms.

**Table 4:** Security comparison of revocable schemes.

Schemes	Underlying Assumption	Quantum Resistant	Security Model
**[20]**	DBDH	×	IND-CPA
**[19]**	DBDH	×	IND-CPA
**[22]**	LWE	✓	IND-sID-CPA
**[15]**	LWE	✓	IND-ID-CPA
**Our**	LWE	✓	IND-sID-CPA

**Table 5:** Functionality comparison of revocable schemes.

Schemes	Type	Multi-bit	Direct Revocable	Collusion Resistant	KGC Overhead
**[20]**	AB-PRE	✓	✓	✓	–
**[19]**	IB-PRE	✓	×	✓	*O*(*N*)
**[22]**	IBE	×	×	×	O(rlog(N/r))
**[15]**	IB-PRE	×	×	✓	O(rlog(N/r))
**Our**	IB-PRE	✓	✓	✓	0

**Table 6:** Theoretical comparison of Identity-based Revocable schemes.

Schemes	The size of secret key	CT	RL authority
**[22]**	O(logN)	*O*(1)	KGC
**[15]**	O(logN)	*O*(1)	KGC
**[43]**	O(logN)	*O*(1)	KGC
**Our**	*O*(1)	O(rlog(N/r))	Encryptor

**Table 7:** Computational overhead of Identity-based Revocable proxy re-encryption schemes.

Step	[15]	Our
Key generation	$3T_{\text{SL}}+1T_{\text{ExtRnd}}$	$1T_{\text{SBL}}+1T_{\text{SP}}$
Encryption	2*T*_Mvmul_	(2 + |*Y*|)*T*_Mvmul_
Re-encryption key generation	$(6mk+1)T_{\text{Hs\_sign}}$	1*T*_SL_
Re-encryption	$1T_{\text{Mvmul}}+1T_{\text{Hs\_Eval}}$	1*T*_Mvmul_
Decryption	1*T*_InnerProduct_	1*T*_InnerProduct_

**Table 8:** Estimated Key and Ciphertext Sizes under Experimental Parameters.

Object	Formula	Estimated Size
MSK (${𝐓}_{{𝐀}_{0}}$)	*m* ^2^	≈2.0 MB
${sk}_{id}$	$4m^{2}+2m\ell$	≈9.0 MB
${rk}_{A\to B,y}$	6*m*^2^	≈12.0 MB
${𝖢}_{{id}_{A}}$	(2+Y)m+ℓ	≈24.5 KB (*Y* = 10)
${𝖢}_{{id}_{B}}$	ℓ+2mdY	≈160.5 KB (*d* = 4,*Y* = 10)

**Table 9:** Computational overhead under different numbers of users.

Algorithm	50	100	200	500	1000
Setup (ms)	204.63	209.88	215.13	222.07	227.32
KeyGen (ms)	189.16	253.94	316.15	389.11	484.68
Encrypt (ms)	31.69	31.99	32.29	32.69	32.99
ReKeyGen (ms)	97.47	102.70	107.20	110.17	115.42
ReEncrypt (ms)	18.98	20.24	24.51	30.85	35.12
Decryption (ms)	3.08	3.10	3.11	3.13	3.15

Moreover, we conduct simulation experiments for the proposed scheme and representative related schemes under the same experimental setting. The experiments are performed on a machine equipped with an NVIDIA RTX 2080Ti GPU, an Intel Core i7-10510U CPU, and 16 GB of RAM. The implementation is written in C++ using the NTL library for modular arithmetic and matrix operations. Microsoft Visual Studio 2022 is used as the compilation and execution environment. The experimental parameters are set as *n* = 32 and *q* = 65536. We have *m* = 256. The revocation-related parameters are set as *N* = 50,100,200,500,1000 and ℓ=256. For the storage estimates, Y=|KUNodes(BT,RL)|=10 denotes the number of covering nodes generated by the complete subtree algorithm, and d=|Path(id)|=4 denotes the length of the identity path. The reported running time is given as the average over these 10 executions.

As shown in Table 5, the proposed IBPRE-DR scheme provides a richer set of functionalities than existing related schemes. Specifically, our construction simultaneously supports identity-based proxy re-encryption, multi-bit message encryption, direct user revocation, and collusion resistance. In contrast, schemes [22] and [15] do not support multi-bit encryption or direct revocation, while scheme [19] relies on indirect revocation mechanisms. Moreover, unlike existing revocable schemes that require periodic key-update generation and distribution by the KGC, the proposed scheme eliminates KGC update overhead by allowing the data owner to directly manage the revocation list during encryption. Therefore, the proposed scheme achieves more flexible and timely access control for cloud data sharing environments.

Table 4 compares the security properties of the proposed scheme with representative related works. It can be observed that schemes [19] and [20] are constructed under traditional hardness assumptions and therefore do not provide resistance against quantum adversaries. By contrast, schemes [22], [15], and the proposed scheme are based on the LWE assumption, which is widely regarded as a standard foundation for post-quantum cryptography. Furthermore, the proposed IBPRE-DR scheme achieves IND-sID-CPA security under the LWE assumption while maintaining support for direct revocation and collusion resistance. Consequently, the proposed construction offers a stronger combination of security guarantees and practical functionality for post-quantum cloud data sharing applications.

Table 6 compares the proposed scheme with representative identity-based revocable schemes in terms of key size, ciphertext size, and revocation authority. As shown in the table, our scheme keeps the user secret key size constant and shifts the revocation workload from the KGC to the encryptor, while the ciphertext size increases with the covering node set.

The computational efficiency of our scheme relative to Wang’s revocable proxy re-encryption scheme is shown in Table 7. *T*_SP_, *T*_SBL_, *T*_SL_, and *T*_ExtRnd_ denote the time consumed by the algorithms SamplePre, SampleBasisLeft, SampleLeft, and ExtRndLeft, respectively. *T*_Mvmul_ represents the time consumed by matrix-vector multiplication operations performed in the group ℤ. $T_{\text{Hs\_Eval}}$ and $T_{\text{Hs\_Sign}}$ denote the time for homomorphic signature generation and homomorphic signature evaluation, respectively. Here, |Y|=rlog(N/r).

**Qualitative discussion and trade-offs.** The comparisons in Table 4–7 show that different schemes achieve different balances among security, functionality, and efficiency. Classical pairing-based PRE or ABPRE schemes can support flexible access control and revocation, but their security relies on traditional hardness assumptions and therefore they are not resistant to quantum attacks. In contrast, lattice-based schemes provide a promising post-quantum foundation, but many existing lattice-based revocable IBE or ABE schemes do not support proxy re-encryption, which limits their applicability in encrypted cloud data sharing scenarios where ciphertext transformation is required.

Compared with indirect revocation schemes, the main advantage of the proposed IBPRE-DR scheme is that revocation is enforced directly by the data owner during encryption. Therefore, the KGC does not need to periodically generate and distribute key-update information for non-revoked users. This design improves revocation timeliness and reduces the online management burden of the KGC. Moreover, the proposed scheme simultaneously supports identity-based proxy re-encryption, multi-bit encryption, direct revocation, and collusion resistance under the LWE assumption, making it suitable for post-quantum cloud data sharing environments with dynamic user membership.

However, these advantages come with certain trade-offs. Since the proposed scheme uses a complete subtree revocation mechanism, the ciphertext contains components associated with the covering set KUNodes(BT,RL). As a result, the ciphertext size and part of the encryption/re-encryption overhead depend on the number of covering nodes. In general, the size of this covering set is related to the number of revoked users and the total number of users, and is bounded by O(rlog(N/r)), where r=|RL|. Therefore, when the system scale *N* is very large or the number of revoked users increases significantly, the ciphertext size and revocation-related computation may also increase.

Thus, the proposed scheme trades a moderate increase in ciphertext and revocation-management overhead for direct revocation, reduced KGC update burden, collusion resistance, and post-quantum security. It is particularly suitable for cloud storage scenarios in which user membership changes dynamically and revoked users should be excluded immediately without waiting for periodic key updates. For extremely large-scale deployments, further optimization of the revocation structure or the adoption of more compact lattice assumptions would be meaningful future research directions.

We estimate the storage costs under the experimental parameter setting used in our implementation: Here, Y=|KUNodes(BT,RL)| denotes the number of non-revoked covering nodes, and d=|Path(id)| denotes the length of the identity path. The values in Table 8 reflect the actual storage overhead in our implementation and are used for the experimental evaluation in Section 4.3.

Table 9 reports the average running time of each algorithm in the proposed IBPRE-DR scheme under different numbers of system users *N*. Each value is the average of 10 executions.

**Fig 4:**
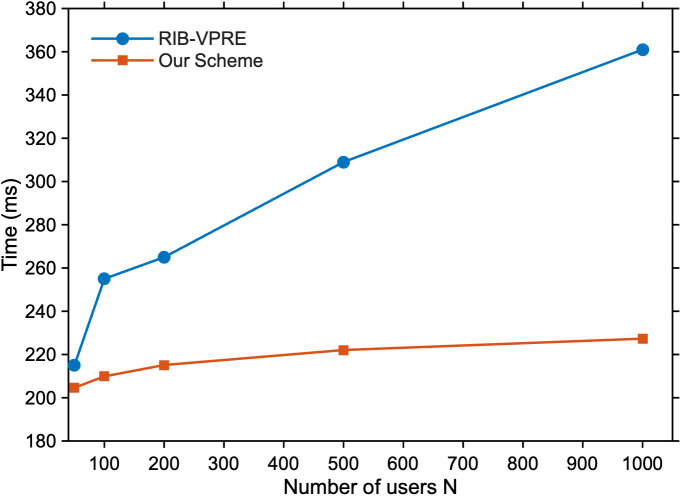
Time for System initialization.

**Fig 5:**
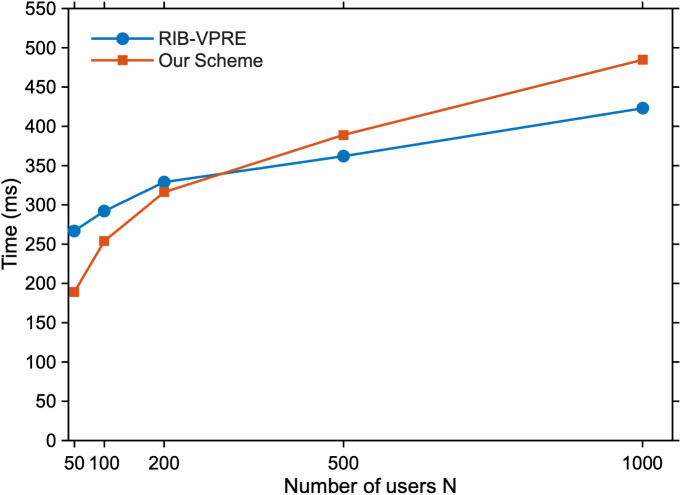
Time for KeyGen Generation.

**Fig 6:**
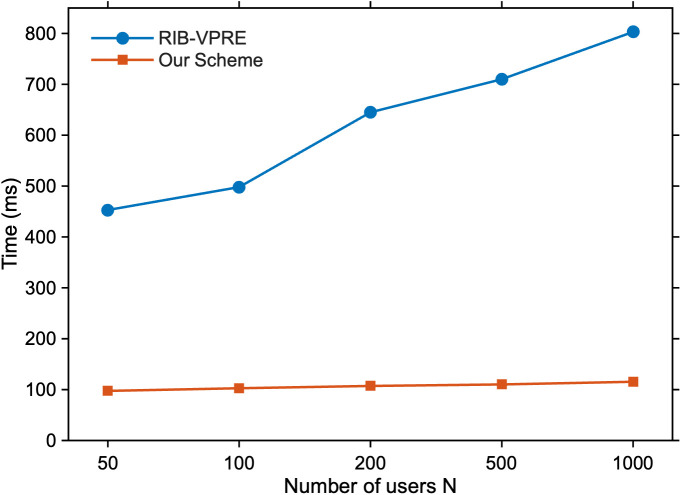
Time for ReKeyGen Generation.

**Fig 7:**
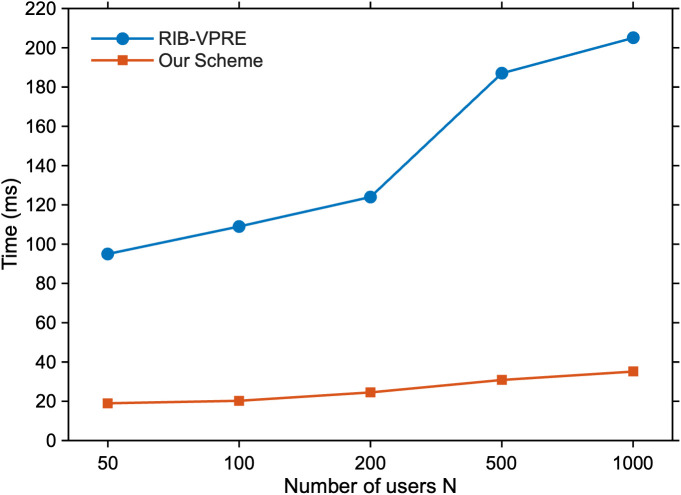
Time for ReEncrypt.

Fig 4, Fig 5, Fig 6, Fig 7 illustrates the time costs of system setup, Key Generation, re-encryption key generation, and re-encryption. Where RIB-VPRE is [15].

As depicted in Fig 4, the system setup procedure exhibits a time cost that grows in direct proportion to the total user count. This is because the initialization process requires generating and assigning binary tree nodes and associated keys for each user. While the time cost grows in proportion to the user count, the slope is relatively gentle, making the overhead acceptable even in large-scale systems. When contrasted with existing indirect revocation schemes built on lattices, our scheme does not require pre-generating a large number of update keys during the setup phase.

Fig 5, The computational overhead of the RIB‑VPRE scheme increases in an approximately linear fashion with a modest slope, indicating that its dominant operations scale directly with the number of users while maintaining low per‑user cost. In contrast, our scheme achieves higher efficiency for small‑scale systems by circumventing the pre‑generation of numerous update keys. However, as the user population expands, the cumulative overhead arising from binary‑tree node management, matrix‑vector multiplications, and path‑based key derivation grows more rapidly, causing the time cost of our scheme to exceed that of RIB‑VPRE in large‑scale settings. Nevertheless, for medium‑scale deployments (N≤500), our scheme still delivers performance that is comparable to, or even better than, that of RIB‑VPRE.

Fig 6 depicts the time cost of re-encryption key generation. The time needed for re-encryption key generation scales logarithmically with respect to the total user count. This is due to the use of a binary tree structure for managing user revocation, where key generation only needs to cover the path nodes related to non-revoked users rather than all users.

As shown in Fig 7, the computational overhead incurred during the re-encryption phase remains largely unaffected with respect to the total user count. Instead, it is primarily influenced by the structure of the ciphertext and the computational complexity corresponding to the re-encryption key. Our scheme’s re-encryption operation involves only matrix-vector multiplications and a small amount of noise operations, resulting in low computational overhead. In contrast, some existing schemes require complex homomorphic operations or multiple key derivations during re-encryption, leading to time costs which grow as the user base expands.

### 4.4. Discussion on the choice of the LWE assumption

The proposed IBPRE-DR scheme is constructed under the standard Learning With Errors (LWE) assumption. We acknowledge that Ring-LWE (RLWE) and Module-LWE (MLWE) are generally considered more efficient in practical implementations due to their compact key and ciphertext representations as well as the availability of fast polynomial arithmetic techniques such as the Number Theoretic Transform (NTT).

However, the primary objective of this work is not to optimize the underlying lattice representation but to realize an efficient identity-based proxy re-encryption scheme with direct revocation. The proposed construction relies on GPV-style trapdoor delegation techniques, including TrapGen, SampleBasisLeft, SamplePre, and SampleLeft, which are naturally defined in the matrix-lattice setting and provide a flexible framework for identity embedding, re-encryption key generation, and binary-tree-based revocation management.

Moreover, the efficiency improvements achieved by the proposed scheme mainly originate from the direct revocation mechanism. Unlike traditional revocable lattice-based constructions that require periodic key-update generation and distribution by the KGC, our scheme allows the encryptor to directly specify the revocation list during encryption. As a result, the workload of the KGC and the communication overhead associated with key updates are significantly reduced.

Therefore, while RLWE and MLWE based constructions may offer superior low-level efficiency in terms of key size and arithmetic operations, the efficiency gains claimed in this work are primarily achieved at the protocol level through the elimination of update-key management and the simplification of revocation operations. Exploring RLWE- or MLWE-based direct revocable proxy re-encryption schemes remains an interesting direction for future research.

### 4.5. Discussion

Discussion on Formal Verification Tools. Formal verification tools such as AVISPA and ProVerif are widely used to analyze security protocols under symbolic security models, particularly for verifying secrecy and authentication properties in message-exchange protocols [44–46]. These tools are based on symbolic abstractions of cryptographic primitives and are effective for detecting attacks such as replay, impersonation, and man-in-the-middle attacks in protocol executions.

However, the proposed IBPRE-DR scheme is a lattice-based identity-based proxy re-encryption construction whose security is defined through IND-sID-CPA indistinguishability and proven by reduction to the hardness of the Learning With Errors (LWE) problem [47–49]. Such computational indistinguishability-based security properties and lattice hardness assumptions cannot be directly verified by symbolic protocol verification tools such as AVISPA. Therefore, the primary security guarantee of the proposed scheme is established through the game-based cryptographic proof presented in this paper.

Nevertheless, automated symbolic verification remains valuable for protocol-level authentication or key-establishment layers that may be built on top of the proposed encryption scheme. Extending the proposed framework with such an authenticated protocol layer and verifying it using tools such as AVISPA or ProVerif is an interesting direction for future work.

## 5. Limitations and future work

Although the proposed IBPRE-DR scheme achieves direct revocation and post-quantum security under the standard LWE assumption, some limitations remain. Since the construction is based on matrix-based LWE, its public parameters, private keys, and ciphertexts may be larger than those of schemes built from more compact structured lattice assumptions, such as Ring-LWE (RLWE) and Module-LWE (MLWE). Therefore, designing RLWE- or MLWE-based variants of the proposed scheme is an important direction for future work.

In addition, the current scheme focuses mainly on encryption, re-encryption, and revocation mechanisms. In future work, we will consider integrating authentication mechanisms into the framework and using formal verification tools such as AVISPA to analyze extended system-level protocols that combine authentication, encryption, re-encryption, and revocation.

## 6. Conclusion

To the best of our knowledge, IBPRE-DR is the first lattice-based identity-based proxy re-encryption scheme that integrates direct user revocation under the standard LWE assumption. We have shown that IBPRE-DR is IND-sID-CPA secure in the standard model and resistant to quantum attacks. During the key generation stage, a dual-key approach is employed, providing each user with two keys for re-encryption key generation and ciphertext decryption, respectively. Even under collusion involving the proxy and an authorized data user, they cannot derive the data owner’s private key from their partial private keys, thereby achieving collusion resistance. Furthermore, this scheme supports multi-bit encryption and incorporates an efficient revocation mechanism. By allowing the data owner to specify the revocation list during encryption, the scheme eliminates the need for the KGC to periodically refresh keys for non-revoked users. This reduces the KGC update overhead from O(rlog(N/r)) to nearly zero and removes the communication cost and latency associated with key updates. This enables immediate revocation and makes the scheme effective for cloud storage environments with dynamic user populations.
